# Four New Flavonoids Isolated from the Aerial Parts of *Cadaba rotundifolia* Forssk. (Qadab)

**DOI:** 10.3390/molecules24112167

**Published:** 2019-06-09

**Authors:** Gadah Abdulaziz Al-Hamoud, Raha Saud Orfali, Sachiko Sugimoto, Yoshi Yamano, Nafee Alothyqi, Ali Mohammed Alzahrani, Katsuyoshi Matsunami

**Affiliations:** 1 Department of Pharmacognosy, Graduate School of Biomedical and Health Sciences, Hiroshima University, 1-2-3 Kasumi, Minami-ku, Hiroshima 734-8553, Japan; galhamoud@ksu.edu.sa (G.A.A.-H.); ssugimot@hiroshima-u.ac.jp (S.S.); yamano@hiroshima-u.ac.jp (Y.Y.); 2 Department of Pharmacognosy, College of Pharmacy, King Saud University, 11495 Riyadh, Saudi Arabia; 3 Department of Biology, Umm Al-Qura University, 1109 Makkah Al-Mukarramah, Saudi Arabia; niothyqi@uqu.edu.sa; 4 Department of Biology, Arts and Sciences in Qilwah, Al-Baha University, 1988 Al-Baha, Saudi Arabia; alialzahrani@bu.edu.sa

**Keywords:** *Cadaba rotundifolia*, Capparaceae, Qadab, aerial parts, flavonoid, antioxidant, AGEs

## Abstract

*Cadaba rotundifolia* (Forssk.) (family: Capparaceae; common name: Qadab) is one of four species that grow in the Red Sea costal region in the Kingdom of Saudi Arabia. The roots and leaves of *C. rotundifolia* is traditionally used to treat tumors and abscesses in Sudan. A previous phytochemical study of the roots yielded a quaternary alkaloid, but no report on chemical constituents of the aerial parts of the *C. rotundifolia* growing in Saudi Arabia has been issued so far. Oxidative stress and advanced glycation end products (AGEs) are thought as causal factors in many degenerative diseases, such as Alzheimer’s disease, diabetes, atherosclerosis and aging. In this study, a total of twenty compounds, including four previously undescribed acylated kaempferol glucosides, were isolated from the aerial parts of *C. rotundifolia* collected in Saudi Arabia. These new compounds were identified as kaempferol 3-*O*-[2-*O*-(*trans*-feruloyl)-3-*O*-β-d-glucopyranosyl]-*β*-d-glucopyranoside (**1**), kaempferol 3-*O*-β-neohesperidoside-7-*O*-[2-*O*-(*cis*-*p*-coumaroyl)-3-*O*-β-d-glucopyranosyl]-β-d-glucopyranoside (**2**), kaempferol 3-*O*-[2,6-di-*O*-α-l-rhamnopyranosyl]-β-d-glucopyranoside-7-*O*-[6-*O*-(*trans*-feruloyl)]-β-d-glucopyranoside (**3**) and kaempferol 3-*O*-[2,6-di-*O*-α-l-rhamnopyranosyl]-β-d-glucopyranoside-7-*O*-[6-*O*-(*trans*-*p*-coumaroyl)]-β-d-glucopyranoside (**4**). Their structures were established based on UV-visible, 1D, 2D NMR, and HR-ESI-MS analyses. Of the assayed compounds, **17** and **18** showed potent radical scavenging activity with IC_50_ values of 14.5 and 11.7 µM, respectively, and inhibitory activity toward AGEs together with compound **7** with IC_50_ values 96.5, 34.9 and 85.5 µM, respectively.

## 1. Introduction

Oxidative stress and advanced glycation end products (AGEs) are thought as causal factors of many diseases, such as diabetes, atherosclerosis, neurodegenerative disorders and aging. Therefore, the discovery of antioxidant and anti-AGEs molecules are important for preventing these age-related pathogenesis. *Cadaba* (Forssk.) is one of genera in the Capparaceae family, with about 30 species distributed in southern Africa, India, Malaysia, and Australia [1,2]. Among them four species grow in the Red Sea costal region in the Kingdom of Saudi Arabia [3]. Phytochemical studies of several *Cadaba* species led to isolation of various secondary metabolites, such as alkaloids, terpenoids and flavonoids [4,5,6,7]. *Cadaba rotundifolia* Forssk., known by the common name Qadab, is an erect shrub with numerous drooping branches with young twigs covered with short glandular hair [8]. In Sudan, *C. rotundifolia* roots and leaves are traditionally used to treat tumors and abscesses. A previous chemical study of the ethanolic extract of *C. rotundifolia* root yielded a quaternary alkaloid (3-hydroxystachydrine) [9]. However, no report on chemical constituents of the aerial parts of *C. rotundifolia* has been issued so far. In this work, we have investigated the chemical profile of the aerial parts of *C. rotundifolia* collected in Saudi Arabia. As a result, twenty flavonoids were obtained, including four undescribed acylated kaempferol glucosides **1**–**4** and 16 known compounds. This paper describes on the isolation, structural elucidation, and evaluation of antioxidant (DPPH) and inhibitory activity toward AGEs formation.

## 2. Results and Discussion

### 2.1. Isolation and Spectroscopic Analyses of the Compounds

The aerial parts of *C. rotundifolia* were extracted with MeOH at room temperature till exhausted. The MeOH extract (800 g) was further separated by solvent fractionation, various column chromatographies (CC), and HPLC to afford compounds **1**–**20** (Figure 1). The chemical structures of the isolated compounds were investigated and identified through intensive spectroscopic analyses based on UV-visible, 1D and 2D NMR, and HR-ESI-MS data as follows (Figures S1–S35).

#### 2.1.1. Chemical Structure of Compound **1**

**Figure 2:**
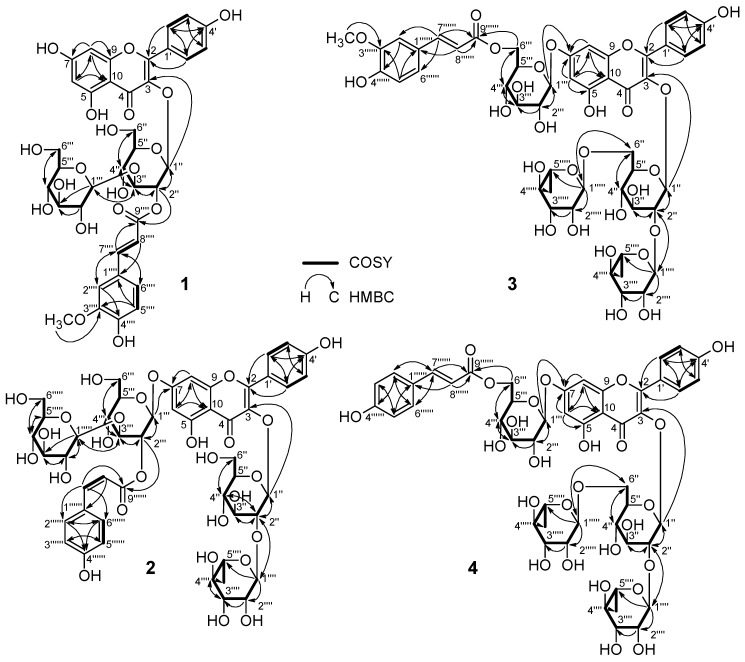
COSY and HMBC correlations of **1**–**4**.

Compound **1** was isolated as a yellow amorphous powder, soluble in MeOH with a negative optical rotation ([α]^22^_D_ −79.0). Its molecular formula, C_37_H_38_O_19_, was determined by positive HR-ESI-MS analysis (*m*/*z* 809.1888 [M + Na]^+^,calcd for C_37_H_38_O_19_Na 809.1899). It had a UV spectrum typical of an acylated kaempferol glycoside (λ_max_ 327, 267 nm) [10,11]. The IR spectrum suggested the presence of hydroxy (3388 cm^−1^), α,β-unsaturated carbonyl ester (1710 cm^−1^), α,β-unsaturated ketone (1658 cm^−1^), aromatic ring (1513, 1446 cm^−1^) and ether (1176, 1070 cm^−1^) functions. The proton and carbon resonances in its ^1^H and ^13^C-NMR spectra clarified the aromatic and glycosidic nature of **1**. It was found that the ^1^H-NMR spectrum of **1** displayed feature resembling to those of **5** [12], as both the aglycone and glycoside units were almost identical. The major differences were in the signals related to the acyl group of **5**. The ^1^H-NMR spectrum of **1** (Table 1) exhibited *meta*-coupled resonances at δ_H_ 6.17 and 6.37 (each 1H, d, *J =* 2.0 Hz), characteristic of H-6 and H-8 of the A-ring, respectively. The ^1^H–^1^H correlation spectrum (COSY, Figure 2) and ^1^H-NMR spectrum of **1** displayed two *ortho*-coupled doublet resonances at δ_H_ 8.01 and 6.91 (each 2H, d, *J =* 8.8 Hz) assignable to H-2′/6′ and H-3′/5′ of the B-ring, respectively, which indicated the presence of a kaempferol aglycone (Table 1).

The ^1^H-NMR spectrum of **1** (Table 1) also gave signals at δ_H_ 6.83 (1H, d, *J =* 8.2 Hz), 7.09 (1H, d, *J =* 8.2, 1.8 Hz) and 7.20 (1H, d, *J =* 1.8 Hz) attributable to an ABX coupling system for a 1,3,4-trisubstituted aromatic moiety, and two *trans*-coupled olefinic protons at δ_H_ 6.40 (1H, br d, *J =* 15.9 Hz) and 7.68 (1H, br d, *J =* 15.9 Hz), together with a singlet signal for a methoxy group at δ_H_ 3.92 which suggested the presence of a feruloyl or isoferuloyl function. The two anomeric proton resonances, which occur at δ_H_ 5.73 (1H, d, *J =* 8.0 Hz) and 4.43 (1H, d, *J =* 7.8 Hz) and 12 carbon signals (δc 104.9, 100.5, 84.9, 78.4, 78.1 , 77.7, 74.8, 74.6, 71.4, 70.0, 62.5 and 62.4) correspond to two glucose moieties. Acid hydrolysis of **1** released d- glucose and the β-anomeric configuration for the sugars was determined from the magnitude of the J_1,2_ coupling constants. In addition, alkaline hydrolysis afforded ferulic acid, which was identified by comparing with an authentic sample based on their HPLC retention times. The ^13^C-NMR spectrum (Table 2) of **1** showed 37 carbon resonances, 12 of which assigned to two β-glucose units, 15 signals were consistent with kaempferol aglycone and the remaining 10 signals were ascribed to acyl groups. The correlations observed between the proton and carbon resonances of the acyl moiety from H-7′′′′ (δH 7.68) to C-2′′′′ (δc 111.7), C-6′′′′ (δc 124.3) and an ester carbonyl C-9′′′′ (δc 168.5), and from the protons of methoxy group (δ_H_ 3.92) to C-3′′′′ (δc 149.3) in HMBC spectrum (Figure 2) indicated the presence of a feruloyl unit. 

According to the coupling constant of the olefinic protons at H-7′′′′ and H-8′′′′ (*J =* 15.9 Hz), the configuration of the feruloyl moiety was determined as *trans*. Full assignments of the ^1^H and ^13^C resonances of the structural fragments of **1** were achieved by analysis of COSY, HSQC and HMBC spectra. A correlation between the anomeric H-1′′ proton (δ_H_ 5.73) and δc 134.8 in the HMBC spectrum (Figure 2) defined C-3 of kaempferol as a site of O-glucosylation. Moreover, the interglucosidic linkage of the disaccharide moiety was also determined by the HMBC cross-peak between H-3′′ (δ_H_ 3.91) and C-1′′′ (δc 104.9). The *E*-feruloyl unit was linked to C-2′′ based on the correlations of the H-2′′ (δ_H_ 5.23) with C-1′′ (δc 100.5), C-3′′ (δc 84.9) and ester carbonyl carbon C-9′′′′ (δc 168.5). Therefore, the structure of compound **1** was determined as kaempferol 3-*O*-[2-*O*-(trans-feruloyl)-3-*O*-β-d-glucopyranosyl]-β-d-glucopyranoside.

#### 2.1.2. Chemical Structure of Compound **2**

Compound **2** was isolated as a yellow amorphous powder, with a positive optical rotation ([α] ^22^_D_ +14.5). Its molecular formula was determined to be C_48_H_56_O_27_ by positive HR-ESI-MS (*m*/*z* 1087.2875 ([M + Na]^+^, calcd for C_48_H_56_O_27_Na 1087.2901). The UV profile of **2** displayed λ_max_ 316, 267 nm, which was similar to those of the kaempferol glycoside acylated with a hydroxycinnamic acid [10,11,13]. The IR spectrum indicated absorptions of hydroxy (3397 cm^−1^), α,β-unsaturated carbonyl ester (1721 cm^−1^), α,β-unsaturated ketone (1657 cm^−1^), aromatic ring (1512, 1442 cm^−1^) and ether (1157, 1072 cm^−1^) functions. The above features were confirmed by the ^1^H-NMR spectrum which revealed a set of kaempferol signals, a *p*-coumaroyl group and glycoside moieties. The kaempferol aglycone of **2** was represented in the ^1^H-NMR spectrum (Table 1) by two *ortho*-coupled resonances of the B-ring at δ_H_ 8.06 and 6.89 (each 1H, d, *J =* 8.9 Hz), typical of H-2′/6′ and H-3′/5′, respectively, in addition to two *meta*-coupled proton resonances at δ_H_ 6.37 and 6.65 (each 1H, br s), attributed to H-6 and H-8 of the A-ring, respectively. In comparison with literature, the downfield shifts of H-6 and H-8 in the ^1^H-NMR spectrum suggested the presence of substitution at C-7 of kaempferol, while the substitution at C-3 was evident from the downfield shift of C-2 to δ_C_ 159.2 in the ^13^C-NMR spectrum. Moreover, the ^1^H-NMR spectrum (Table 1) exhibited two olefinic protons at δ_H_ 5.83 and 6.86 with a coupling constant of 12.9 Hz. Similarly, the AA′BB′ aromatic coupling system at δ_H_ 6.67 and 7.55 (each 1H, d, *J =* 8.6 Hz) were attributed to a *p*-coumaroyl unit, which was confirmed by the correlation of the olefinic protons (δ_H_ 5.83 and 6.86) with the ester carbonyl carbon at δc 167.2 and aromatic carbons (δ_C_ 127.6; C-1′′′′′′ and 133.4; C-2/6′′′′′′) in the HMBC spectrum (Figure 2). The sugar units in **2** were represented in the ^1^H-NMR spectra by four anomeric proton signals at δ_H_ 4.40 (1H, d, *J =* 7.8 Hz), 5.22 (1H, d, *J =* 1.3 Hz), 5.29 (1H, d, *J =* 7.8 Hz), 5.72 (1H, d, *J =* 7.6 Hz) and one methyl group at 0.96 (3H, d, *J =* 6.18 Hz), which indicated the tetraglycosidic nature of **2**. Identification of the sugar residues was carried out by careful analysis of the 1D and 2D NMR data as well as comparison with the reported chemical shift values. The absolute stereochemistry of the sugars was determined by acid hydrolysis of **2** and HPLC analysis with an optical rotation detector, and the magnitudes of *J*_1,2_ coupling constants that revealed β-d-glucopyranose and α-l-rhamnopyranose moieties. The spectroscopic data of **2** (Table 1 and Table 2) indicated that it is an isomer of **12** [13]. The relatively smaller coupling constant (*J =* 12.9 Hz) and the chemical shift values of H-7′′′′′′ and H-8′′′′′′ (δ_H_ 6.86 and 5.83, respectively) indicated **2** as a *cis* isomer. In addition, alkaline hydrolysis of **2** afforded *cis*-*p*-coumaric acid. The positions of glycosylation and the *cis*-*p*-coumaroyl moiety on the kaempferol aglycone were decided using the HMBC spectrum (Figure 2). The anomeric protons of two glucose moieties at δ_H_ 5.72 and 5.29 have long-range correlations with C-3 (δc 134.7) and C-7 (δc 163.8) of the kaempferol aglycone, respectively, indicating the connectivity of the sugars. The COSY correlation (Figure 2) of H-2′′ at (δ_H_ 3.60) with H-1′′ at (δ_H_ 5.72), together with correlation observed in the HMBC spectrum between H-2′′and C-1′′′′ of rhamnose at (δc 102.6) confirmed the presence of a β-neohesperidoside structure at C-3 of kaempferol. Moreover, the correlations of H-2′′′ (δ_H_ 5.25) with H-1′′′ (δ_H_ 5.29) and H-3′′′ (δ_H_ 3.92) in COSY spectrum, and the correlations from H-2′′′ to C-9′′′′′′ (δc 167.2), and from H-3′′′ to C-1′′′′′ (δc 105.0) in HMBC spectrum confirmed the presence of 7*-O-*[2*-O-*(*cis-p*-coumaroyl)-3*-O-*β-d-gluco-pyranosyl]-β-d-glucopyranose as the kaempferol aglycone. The structure of **2** was therefore identified as kaempferol 3*-O-*β-neohesperidoside-7*-O-*[2*-O-*(*cis-p*-coumaroyl)-3*-O-*β-d-glucopyranosyl]-β-d-glucopyranoside.

#### 2.1.3. Chemical Structure of Compound **3**

Compound **3** was obtained as a yellow amorphous powder with a negative optical rotation ([α]^22^_D_ −82.5). The molecular formula, C_49_H_58_O_27_, was deduced from the sodiated molecular ion peak at *m*/*z* 1101.3021 (calcd for C_49_H_58_O_27_Na 1101.3058) by HR-ESI-MS measurement. It displayed characteristic UV spectrum of an acylated kaempferol glycoside with λ_max_ at 329, 268 nm, similar to those of **1**. The IR spectrum exhibited absorptions of hydroxy (3389 cm^−1^), α,β-unsaturated ketone (1651 cm^−1^), aromatic ring (1513, 1455 cm^−1^) and ether (1180, 1071 cm^−1^) functions. Analysis of the ^1^H- and ^13^C-NMR spectra of **3** (Table 1 and Table 2) indicated the presence of a kaempferol residue, four hexose sugar moieties, and an *E*-feruloyl unit. The ^1^H spectrum (Table 1) displayed four downfield doublet signals at δ_H_ 6.46 (1H, d, *J =* 2.1 Hz), 6.61 (1H, d, *J =* 2.1 Hz), 6.76 (2 H, d, *J =* 8.9 Hz) and 7.93 (2 H, d, *J =* 8.9 Hz) assignable to H-6, 8, 3′/5′ and 2′/6′, respectively, belonging to kaempferol aglycone. Moreover, the characteristic signals of the *E*-feruloyl moiety were observed in ^1^H-NMR spectrum as five downfield doublets at δ_H_ 6.28 (1H, br d, *J =* 15.8 Hz), 6.67 (1H, d, *J =* 8.2 Hz), 6.85 (1H, d, *J =* 8.2, 1.7 Hz), 6.98 (1H, d, *J =* 1.7 Hz) and 7.50 (1H, br d, *J =* 15.8 Hz), corresponding to H-8′′′′′′, 5′′′′′′, 6′′′′′′, 2′′′′′′ and 7′′′′′′, respectively, in addition to a singlet signal of a methoxy group at δ_H_ 3.75. Full assignment of the 1D NMR spectra of **3** was achieved by analysis of COSY, HSQC and HMBC data, which confirmed presence of a 3,7*-O-*glycosidic kaempferol residue together with the *E*-feruloyl unit. Acid hydrolysis of **3** with 1 M HCl released d-glucose and l-rhamnose moieties, while alkaline hydrolysis afforded a ferulic acid moiety, identified by HPLC comparison with authentic samples. The ^1^H-NMR spectrum of **3** exhibited resonances for anomeric protons of O-linked sugars at δ_H_ 4.98 (1H, d, *J =* 7.4 Hz) and 5.51 (1H, d, *J =* 7.6 Hz), corresponding to two d-glucoses, and 4.38 (1H, d, *J =* 1.3 Hz) and 5.14 (1H, d, *J =* 1.1 Hz), together with typical doublets of two methyl groups at δ_H_ 0.88 (3H, d, *J =* 6.2 Hz) and 0.96 (3H, d, *J =* 6.2 Hz), corresponding to two l-rhamnose moieties. Full assignment of the ^1^H and ^13^C-NMR data of each sugar was achieved using standard 2D NMR experiments (Table 1 and Table 2). The configurations of these sugars were identified from the magnitude of their *J*_1,2_ coupling constants and the chemical shifts as β-d-glucopyranose and α-l-rhamnopyranose. The glycosidation sites on the kaempferol aglycone of **3** were determined from the long-range correlations exhibited in the HMBC spectrum from H-1′′ δ_H_ 5.51 to C-3 (δc 134.6), and from H-1′′′ 4.98 to C-7 (164.4). Both C-2′′ and C-6′′ of the β-glucose attached at C-3 were shifted in the ^13^C-NMR spectrum (Table 2) to δc 79.7 and 68.3, respectively, and glycosylated by two α-l-rhamnopyranosyl moieties (Rha I and Rha II, respectively), according to the HMBC correlations (Figure 2), their connectivities were detected by the cross peaks between H-2′′ (δ_H_ 3.52) and C-1′′′′ of Rha I (δc 102.5), and between H_2_-6′′ (δ_H_ 3.27 and 3.71) and C-1′′′′′ of Rha II (δc 102.2). Furthermore, the HMBC correlations from significant downfield shifted protons of H_2_-6′′′ of the β-glucose attached at C-7 at δ_H_ 4.17 and 4.57 to C-9′′′′′′ at (δc 169.1) indicated the position of acylation, thus defining the *E*-feruloyl linkage site. Therefore, the structure of compound **3** was elucidated as kaempferol 3*-O-*[2,6-di*-O-*α-l-rhamnopyranosyl]-β-d-glucopyranoside-7*-O-*[6*-O-*(*trans*-feruloyl)]-β-d-glucopyranoside.

#### 2.1.4. Chemical Structure of Compound **4**

Compound **4** was isolated as a yellow amorphous powder, with a negative optical rotation ([α]^22^_D_ −81.6). Its molecular formula was established as C_48_H_56_O_26_ by its positive HR-ESI-MS spectrum (*m*/*z* 1071.2925 [M + Na]^+^, calcd for C_48_H_56_O_26_Na 1071.2952), suggesting the lack of one methoxy group compared to **3**. Analysis of 2D experiments of **4** including COSY, HSQC and HMBC, and comparison of its ^1^H- and ^13^C-NMR values with those of **3** (Table 1 and Table 2) showed that **4** was a kaempferol tetraglycoside acylated with a cinnamic acid derivative. The ^1^H- and ^13^C-NMR spectra of **4** revealed an identical glycosylation profile to that of **3**. Comparison of the HMBC correlations of **4** with those of **3** (Figure 2) detected the same pattern of connectivity defining the linkages between the glycosyl moieties and the kaempferol residue, and between the glycosyl moieties themselves. Thus, the difference between **3** and **4** must be located at the acyl substituent. In particular, the ^1^H-NMR spectrum (Table 1) showed the typical coupling pattern (AA′BB′ system) of a 1,4-disubstituted aromatic moiety at δ_H_ 6.66 and 7.25 (each 2H, d, *J =* 8.6 Hz) and two *trans*-coupled double bond protons at δ_H_ 6.24 and 7.50 (each 1H, br d, *J =* 15.9 Hz), as well as to an ester carbonyl (δc 169.1) in the ^13^C-NMR spectrum assignable to a *p*-coumaroyl unit. In the HMBC spectrum (Figure 2), the cross peaks between H_2_-6′′′ (δ_H_ 4.18 and 4.54) and C-9′′′′′′ (δc 169.1) placed the *p*-coumaroyl unit at C-6′′′.The sugar units were identified as D-glucose and l-rhamnose by acid hydrolysis of **4**, followed by HPLC analysis with chiral detector in comparison with authentic samples. Anomeric configurations were determined from the magnitude of the *J*_1,2_ coupling constants in the ^1^H-NMR spectrum. Therefore, the structure of **4** was a *trans-p*-coumaroylated kaempferol tetraglycoside, i.e., kaempferol 3*-O-*[2,6-di*-O-*α-L-rhamnopyranosyl]-β-d-glucopyranoside-7*-O-*[6*-O-*(trans-p-coumaroyl)]-β-d-gluco-pyranoside.

### 2.2. Biological Activities of the Isolated Compounds

Isolated compounds **1**–**20** from the *n*-BuOH fraction of *C. rotundifolia* were evaluated for DPPH radical scavenging assay activity (Table 3) using Trolox as a positive control (IC_50_: 29.2 ± 0.39 µM). The results showed that compounds **17** and **18** exhibited potent radical scavenging activity with IC_50_ values of 14.5 and 11.7 µM, respectively. On the other hand, compounds **15** and **16** showed moderate activity with IC_50_ values of 43.0 and 31.8 µM, respectively. These results are consistent with previous studies on the effectiveness of myricetin as a potent antioxidant agent which could prevent and slow the progress of ageing or various diseases associated with oxidative stress.

Inhibitory effects of isolated compounds (**1**–**20**) on AGEs formation were also evaluated using a fluorescence method [14]. As shown in Table 3, compound **7** (IC_50_: 85.5 ± 3.5 µM), **17** (IC_50_: 96.5 ± 1.8 µM) and **18** (IC_50_: 34.9 ± 1.2 µM) showed strong inhibitory activity than that of a positive control, aminoguanidine hydrochloride (IC_50_: 7818 ± 34.4 µM). The results revealed that the most inhibitory effect was exhibited by myricetin (**18**). Comparison of IC_50_ values of myricetin (**18**) and its tri-, di-, and mono-glycosides (**15**, **16**, and **17**, respectively) indicated that the number of sugar moieties at C-3 of myricetin aglycone decreases the inhibition activity of AGEs formation. The other compounds did not show any inhibitory activity.

## 3. Materials and Methods

### 3.1. General Methods

Optical rotations were measured on a JASCO P-1030 polarimeter (Jasco, Tokyo, Japan). UV and IR spectra were obtained on a Jasco V-520 UV/Vis spectrophotometer and a Horiba FT-710 Fourier transform infrared spectrophotometer (Horiba, Kyoto, Japan), respectively. NMR experiments were measured on a Bruker Avance 500, 600 and 700 MHz spectrometers (Bruker, Billerica, MA, USA), with tetramethylsilane (TMS) as an internal standard. Positive ion HR-ESI-MS spectra were recorded using a LTQ Orbitrap XL mass spectrometer (Thermo Fisher Scientific, Waltham, MA, USA). Column chromatography (CC) was performed on Diaion HP-20 (Mitsubishi Chemical Corp., Tokyo, Japan), silica gel 60 (230–400 mesh, Merck, Darmstadt, Germany), and octadecyl silica (ODS) gel (Cosmosil 75C_18_-OPN (Nacalai Tesque, Kyoto, Japan; Φ = 35 mm, L = 350 mm), and TLC was performed on precoated silica gel plates 60 GF_254_ (0.25 mm in thickness, Merck). HPLC was performed on ODS gel (Inertsil ODS-3, GL-science, 10 mm × 250 mm, flow rate 2.5 mL/min) with a mixture of H_2_O, acetone and MeOH, and the eluate was monitored by refractive index and/or a UV detector. An amino column (Shodex Asahipak NH2P-50 4E (4.6 mm × 250 mm), CH_3_CN-H_2_O (3:1) 1 mL/min) together with a chiral detector (Jasco OR-2090plus) was used for HPLC analysis of sugars obtained after hydrolysis [15].

### 3.2. Plant Material

Aerial parts of *Cadaba rotundifolia* were collected from eastern region, near Makkah, Saudi Arabia, in April 2017. The collected plant was identified and classified by a plant taxonomist at Biology Department–Umm Al-Qura University, Makkah, Saudi Arabia. A voucher specimen (No. 15501) was deposited in the Herbarium of the Department of Pharmacognosy, Pharmacy College, King Saud University.

### 3.3. Extraction and Isolation

The air-dried powdered aerial parts (6.0 Kg) of *C. rotundifolia* Forssk. were extracted with MeOH (10 L × 7) and then concentrated under reduced pressure to give a viscous gummy material (800 g). This residue was suspended in 900 mL of distilled water and fractionated by shaking with petroleum ether (bp. 60–80 °C) (3L × 5, 132.9 g), CH_2_Cl_2_ (3 L × 5, 6 g), EtOAc (3 L × 5, 10.9 g) and *n*-BuOH (3 L × 5, 118 g), respectively. Part of *n*-BuOH fraction (112 g) was fractionated by Diaion HP-20 column chromatography (Φ = 6 cm, L = 35 cm, 900 g). The column was eluted initially with H_2_O, then with a MeOH/H_2_O stepwise gradient with increasing MeOH content (20, 40, 60, 80, 100% MeOH, respectively, 3 L each), yielding five fractions (Frs. Cr1–Cr5). The fraction Cr2 (10.7 g) was subjected to silica gel column chromatography (Φ = 3 cm, L = 15 cm, 100 g) eluting with increasing amount of MeOH in CHCl_3_ (20:1, 10:1, 7:1, 5:1, 3:1, 1:1, finally with 100% MeOH, 500 mL each) yielding seven fractions (Frs. Cr2.1–Cr2.7). Fraction Cr2.4 (3.5 g) was subjected to open reversed phase (ODS) column chromatography with a linear gradient of 10% aq. MeOH (400 mL)–100% methanol (400 mL), affording eight fractions (Frs. Cr2.4.1–Cr2.4.8). The residue of fraction Cr2.4.4 (187 mg) was purified by preparative HPLC using 46% aq. acetone to give **18** (5.0 mg) and **20** (25.1 mg). Fraction Cr2.5 (2.4 g) was subjected to ODS column chromatography eluting with a linear gradient of 10% aq. MeOH (400 mL)–100% MeOH (400 mL), to give seven fractions (Frs. Cr2.5.1–Cr2.5.7). The residue of fraction Cr2.5.4 (554 mg) was purified by preparative HPLC (25% aq. acetone) to give **6** (4.2 mg). Fraction Cr2.7 (2.6 g) was subjected to ODS column chromatography (linear gradient of 10% aq. MeOH (400 mL)–100% MeOH (400 mL), giving six fractions (Frs. Cr2.7.1–Cr2.7.6). The residue of fraction Cr2.7.2 (632 mg) was purified by preparative HPLC (25% aq. acetone) to give **15** (5.6 mg) and **16** (11.0 mg). Then residue of fraction Cr2.7.3 (1.2 g) was also purified by preparative HPLC (28% aq. acetone) to give **7** (17.3 mg) and **19** (9.2 mg). Fraction Cr3 (15.5 g) was subjected to silica gel column chromatography (Φ = 3 cm, L = 20 cm, 150 g) with increasing amounts of MeOH in CHCl_3_ (20:1, 10:1, 7:1, 5:1, 3:1, 1:1, then 100% MeOH, 400 mL each), yielding seven fractions (Frs. Cr3.1–Cr3.7). Fraction Cr3.3 (2.9 g) was subjected to ODS column chromatography (linear gradient of 10% aq. MeOH (400 mL)–100% MeOH (400 mL), affording nine fractions (Frs. Cr3.3.1–Cr3.3.9). The residue of fraction Cr3.3.4 (645 mg) was purified by preparative HPLC (28% aq. acetone) to give **10** (30.1 mg). Fraction Cr3.4 (2.8 g) was subjected to ODS column chromatography (linear gradient of 10% aq. MeOH (400 mL)–100% MeOH (400 mL)) leading to seven fractions (Frs. Cr3.4.1–Cr3.4.7). The residue of fraction Cr3.4.2 (1.2 g) was purified by preparative HPLC (28% aq. acetone) to give **17** (15.2 mg). The residue of fraction Cr3.4.4 (187 mg) was also purified by preparative HPLC (40% aq. acetone) to give **1** (7.4 mg) and **5** (2.0 mg). Fraction Cr3.5 (4.4 g) was subjected to ODS column chromatography (linear gradient of 10% aq. MeOH (400 mL)–100% MeOH (400 mL)) providing seven fractions (Frs. Cr3.5.1–Cr3.5.7). The residue of fraction Cr3.5.4 (692 mg) was purified by preparative HPLC (28% aq. acetone) to give **8** (39.0 mg) and **9** (20.0 mg). The other residue of fraction Cr3.5.5 (65.7 mg) was purified by preparative HPLC (32% aq. acetone) to give **11** (3.0 mg). Fraction Cr3.6 (760 mg) was subjected to ODS column chromatography (linear gradient of 10% aq. MeOH (400 mL)–100% MeOH (400 mL)), affording seven fractions (Frs. Cr3.6.1–Cr3.6.7). The residue of fraction Cr3.6.4 (923 mg) was purified by preparative HPLC (25% aq. acetone). The following compounds were separated: **2** (5.0 mg), **12** (12.0 mg), **13** (2.0 mg) and **14** (10.0 mg). The residue of fraction Cr3.6.5 (963 mg) was purified by preparative HPLC (35% aq. acetone) to give **3** (7.8 mg) and **4** (2.0 mg).

The known compounds **5**–**20** were identified by comparison of their spectroscopic data with those reported in the literature as kaempferol 3*-O-*[2*-O-*(*trans*-*p*-coumaroyl)-3-*O*-β-d-gluco-pyranosyl]-β-d-glucopyranoside (**5**) [12], kaempferol 3,4′-di-*O*-β-d-glucoside (**6**) [16], kaempferol 3-*O*-[2,6-di-*O*-*α*-l-rhamnopyranosyl]-β-d-glucoside (**7**) [17], kaempferol 3-*O*-β-neohesperidoside (**8**) [17], kaempferol 3-*O*-β-rutinoside (**9**) [17], kaempferol 3-*O*-β-d-glucoside (**10**) [17], rhamnocitrin 3-*O*-β-neohesperidoside (**11**) [18], kaempferol 3-*O*-β-neohesperidoside-7-*O*-[2-*O*-(*trans*-*p*-coumaroyl)-3-*O*-β-d-glucopyranosyl]-β-d-glucopyranoside (**12**) [13], kaempferol 3-*O*-β-neohesperidoside-7-*O*-[2-*O*-(*trans*-feruloyl)]-β-d-glucopyranoside (**13**) [13], kaempferol 3-*O*-β-neohesperidoside-7-*O*-[2-*O*-(*trans*-*p*-coumaroyl)]-β-d-glucopyranoside (**14**) [13], myricetin 3-*O*-[2,6-di-*O*-*α*-l-rhamnopyranosyl]-β-d-glucoside (**15**) [17], myricetin 3-*O*-β-neohesperidoside (**16**) [17], myricetin 3-*O*-β-D-glucoside (**17**) [17], myricetin (**18**) [19], beitingxinhuagtong C (**19**) [20], phloretin (**20**) [21].

### 3.4. Spectroscopic Data of Compounds ***1***–***4***

*Kaempferol 3-O-[2-O-(trans-feruloyl)-3-O-β-d-glucopyranosyl]-β-d-glucopyranoside* (**1**)

Yellow amorphous powder [α]^22^_D_ −79.0 (c 0.42, MeOH); HR-ESI-MS: *m/z*: 809.1888 [M + Na]^+^ (calcd for C_37_H_38_O_19_Na 809.1899); UV λ_max_ (MeOH) nm (log *ɛ*): 327 (4.21), 299 (4.12), 267 (4.13), 231 (4.23); IR (film) ν_max_: 3388, 2936, 1710, 1658, 1604, 1513, 1446, 1363, 1176, 1070, 1028 cm^−1^; ^1^H and ^13^C data see Table 1 and Table 2.

*Kaempferol 3-O-β-neohesperidoside-7-O-[2-O-(cis-p-coumaroyl)-3-O-β-d-glucopyranosyl]-β-d-gluco-pyranoside* (**2**)

Yellow amorphous powder [α]^22^_D_ +14.5 (c 0.60, MeOH); HR-ESI-MS: *m/z*: 1087.2875 [M + Na]^+^ (calcd for C_48_H_56_O_27_Na 1087.2901); UV λ_max_ (MeOH) nm (log *ɛ*): 351 (3.97), 316 (4.14), 267 (4.04), 229 (3.99); IR (film) ν_max_: 3397, 2924, 1721, 1657, 1603, 1512, 1442, 1367, 1157, 1072, 1024 cm^−1^; ^1^H and ^13^C data see Table 1 and Table 2.

*Kaempferol 3-O-[2,6-di-O-α-l-rhamnopyranosyl]-β-d-glucopyranoside-7-O-[6-O-(trans-feruloyl)]-β-d-gluco pyranoside* (**3**)

Yellow amorphous powder [α]^22^_D_ −82.5 (c 1.02, MeOH); HR-ESI-MS: *m/z*: 1101.3026 [M + Na]^+^ (calcd for C_49_H_58_O_27_Na 1101.3058); UV λ_max_ (MeOH) nm (log *ɛ*): 329 (4.17), 295 (4.00), 268 (4.05), 233 (4.11); IR (film) ν_max_: 3389, 2932, 1712, 1651, 1601, 1513, 1455, 1371, 1180, 1071, 1024 cm^−1^; ^1^H and ^13^C data see Table 1 and Table 2.

*Kaempferol 3-O-[2,6-di-O-α-l-rhamnopyranosyl]-β-d-glucopyranoside-7-O-[6-O-(trans-p-coumaroyl)]-β-d-glucopyranoside* (**4**)

Yellow amorphous powder [α]^22^_D_ −81.6 (c 0.36, MeOH); HR-ESI-MS: *m/z*: 1071.2925 [M + Na]^+^ (calcd for C_48_H_56_O_26_Na 1071.2952); UV λ_max_ (MeOH) nm (log *ɛ*): 348 (4.01), 317 (4.15), 267 (4.05), 228 (4.09); IR (film) ν_max_: 3379, 2912, 1660, 1601, 1499, 1445, 1372, 1172, 1074, 1034 cm^−1^; ^1^H and ^13^C data see Table 1 and Table 2.

### 3.5. Acid Hydrolysis

The isolated compounds **1**–**4** (1.0 mg each) were hydrolyzed with 1 M HCl (1.0 mL) at 80 °C for 3 h, then cooled. The reaction mixtures were neutralized with Amberlite IRA96SB (OH^−^ form), then filtered to remove the resin. The filtrates were extracted with EtOAc. The aqueous layers were analyzed by HPLC with an amino column [Asahipak NH2P-50 4E, CH_3_CN-H_2_O (3:1), 1 mL/min] and a chiral detector (JASCO OR-2090plus) in comparison with authentic standards of D-glucose and L-rhamnose. The aqueous layers of the hydrolyzed compounds showed a peak at t_R_ 8.15 min which coincided with that of d-glucose (positive optical rotation). The t_R_ of the l-rhamnose (negative optical rotation) was 6.5 min [15].

### 3.6. Alkaline Hydrolysis

Solutions of **1**–**3** (3.0 mg each) in 50% aqueous 1,4-dioxane (0.5 mL) were treated with 10% aqueous KOH (0.5 mL) and stirred at 37 °C for 1 h. The reaction mixtures were neutralized with an ion-exchange resin Amberlite IR-120B (H^+^-form), then filtered and evaporated. The residues were dissolved in EtOAc and subjected to HPLC analysis [Cosmosil C18-PAQ, MeOH-H_2_O (45:50), 1 mL/min)] with UV (245 nm) detection to identify ferulic acid (t_R_ 16.7 min) from **1** and **3**, and *p*-coumaric acid (t_R_ 15.1 min) from **2** [22]. The authentic *cis*-*p*-coumaric acid was prepared according to the literature [23].

### 3.7. DPPH Radical Scavenging Activity

Free radical scavenging activity were evaluated by using a quantitative DPPH assay. The absorbance of various concentrations of test compounds dissolved in 100 µL of MeOH in 96-well microtiter plate were measured at 515 nm as (A_blank_). Then, a 100 µL of DPPH solution (200 µM) was added to each well. The plate was incubated in dark chamber at room temperature for 30 min before measuring the absorbance (A_sample_) again. The following equation was used to calculate % inhibition of free radicals:

% Inhibition = [1 − (A_sample_ − A_blank_)/(A_control_ − A_blank_)] × 100

where A_control_ is the absorbance of control (DMSO and all reagents, except the test compound). Their IC_50_ values were determined based on three independent experiments. Trolox was used as reference compound [24].

### 3.8. Determination of In Vitro AGEs Formation

The reaction mixture including 0.5 M ribose and 10 mg/mL bovine serum albumin in 50 mM phosphate buffer (pH 7.4)- 0.02% sodium azide was mixed with the test compounds, and incubated for 24 h at 37 °C. The formation of fluorescent AGEs were measured with a fluorometric microplate reader (Ex: 370 nm, Em: 440 nm, EnSpire, PerkinElmer, Inc., Waltham, MA, USA). Experiments were performed in triplicate and IC_50_ values were calculated by linear regression [14].

## 4. Conclusions

In conclusion, four new flavonoid glycosides **1**–**4** together with 16 known compounds **5**–**20** were isolated from the aerial parts of *Cadaba rotundifolia* (Forssk.) (Qadab) collected in Saudi Arabia. The chemical structures of the new compounds were elucidated by intensive spectroscopic analyses and chemical reactions. Among the isolated compounds, **15**–**18** exhibited significant radical scavenging activity and compounds **7**, **17** and **18** showed strong inhibitory activity toward AGEs formation. These results suggest that *C. rotundifolia* and their chemical constituents may ameliorate oxidative stress in various degenerative disorders including Alzheimer’s disease, diabetes, atherosclerosis and aging. Finally, it is noteworthy that most of the known compounds, **5**–**8**, **11**–**16**, **19**, and **20,** were isolated from the family Capparaceae for the first time. The remaining three known compounds, **9**, **10**, **17**, also have not been isolated from the title plant so far. These results indicated that the characteristic circumstances of the oases surrounded by desert in Saudi Arabia provide promising and interesting natural resources for future drug development.

## Figures and Tables

**Figure 1:**
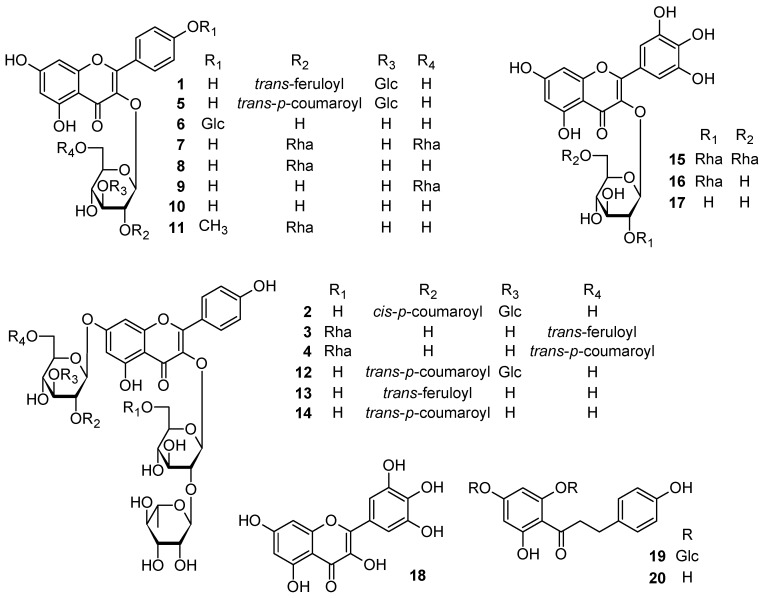
Compounds **1**–**20** isolated from *Cadaba rotundifolia*.

**Table 1:** ^1^H-NMR data of compounds **1**–**4** (600 or 700 * MHz, CD_3_OD, δ in ppm, J in Hz).

Position	1 *	2	3	4	Position	1 *	2	3	4
6	6.17 d (2.0)	6.37 br s	6.46 d (2.1)	6.45 d (2.1)	1′′′′	-	5.22 d (1.3)	5.14 d (1.1)	5.14 br s
8	6.37 d (2.0)	6.65 br s	6.61 d (2.1)	6.62 d (2.1)	2′′′′	7.20 d (1.8)	3.99 m	3.90 m	3.90 m
2′	8.01 d (8.8)	8.06 d (8.9)	7.93 d (8.9)	7.95 d (8.8)	3′′′′	-	3.76 m	3.69 m	3.69 m
3′	6.91 d (8.8)	6.89 d (8.9)	6.76 d (8.9)	6.77 d (8.8)	4′′′′	-	3.33 t (9.6)	3.25 m	3.42 m
5′	6.91 d (8.8)	6.89 d (8.9)	6.76 d (8.9)	6.77 d (8.8)	5′′′′	6.83 d (8.2)	4.02 dq (9.6, 6.18)	3.93 dq (9.5, 6.2)	3.68 dq (9.6, 6.2)
6′	8.01 d (8.8)	8.06 d (8.9)	7.93 d (8.9)	7.95 d (8.8)	6′′′′	7.09 dd (8.2, 1.8)	0.96 d (6.18)	0.88 d (6.2)	0.96 d (6.2)
					7′′′′	7.68 br d (15.9)			
1′′	5.73 d (8.0)	5.72 d (7.6)	5.51 d (7.6)	5.51 d (7.6)	8′′′′	6.40 br d (15.9)			
2′′	5.23 dd (9.5, 8.0)	3.60 m	3.52 dd (9.6, 7.6)	3.52 dd (9.6, 7.6)	OCH_3_	3.92 s			
3′′	3.91 t (9.5)	3.55 t (9.0)	3.45 m	3.44 m					
4′′	3.55 t (9.5)	3.26 t (9.0)	3.13 t (9.12)	3.19 m	1′′′′′		4.40 d (7.8)	4.38 d (1.3)	4.36 br s
5′′	3.39 ddd (9.5, 5.5, 2.0)	3.31 m	3.24 m	3.24 m	2′′′′′		3.20 dd (8.5, 7.8)	3.38 m	3.37 m
6′′	3.62 dd (12.0, 5.5)	3.47 dd (12.0, 5.9)	3.27 m	3.27 m	3′′′′′		3.31 m	3.34 dd (9.4, 3.4)	3.33 dd (9.4, 3.4)
	3.81 dd (12.0, 2.0)	3.71 dd (12.0, 2.1)	3.71 m	3.71 m	4′′′′′		3.29 m	3.12 t (9.4)	3.25 m
					5′′′′′		3.22 m	3.30 m	3.25 m
1′′′	4.43 d (7.8)	5.29 d (7.8)	4.98 d (7.4)	4.98 d (7.3)	6′′′′′		3.87 dd (11.5, 5.4)	0.96 d (6.2)	0.88 d (6.2)
2′′′	3.21 dd (8.8, 7.8)	5.25 dd (9.0, 7.8)	3.42 m	3.41 t (9.4)			3.64 dd (11.5, 1.3)		
3′′′	3.31 t (8.8)	3.92 t (9.0)	3.43 m	3.42 m					
4′′′	3.28 t (8.8)	3.76 m	3.34 m	3.34 t (9.4)	2′′′′′′		7.55 d (8.6)	6.98 d (1.7)	7.25 d (8.6)
5′′′	3.34 m	3.78 m	3.72 m	3.72 m	3′′′′′′		6.67 d (8.6)	-	6.66 d (8.6)
6′′′	3.62 dd (11.9, 2.2)	3.75 dd (12.0, 5.1)	4.17 dd (12.0, 2.0)	4.18 dd (12.0, 2.1)	5′′′′′′		6.67 d (8.6)	6.67 d (8.2)	6.66 d (8.6)
	3.88 dd (11.9, 6.0)	3.94 dd (12.0, 1.6)	4.57 dd (12.0, 6.9)	4.54 dd (12.0, 7.0)	6′′′′′′		7.55 d (8.6)	6.85 d (8.2, 1.7)	7.25 d (8.6)
					7′′′′′′		6.86 br d (12.9)	7.50 br d (15.8)	7.50 br d (15.9)
					8′′′′′′		5.83 br d (12.9)	6.28 br d (15.8)	6.24 br d (15.9)
					OCH_3_			3.75 s	

m: multiplet or overlapped signals.

**Table 2:** ^13^C-NMR data of compounds **1**–**4** (150 or 175 * MHz, CD_3_OD, δ in ppm).

Position	1 *	2	3	4	Position	1 *	2	3	4
2	158.4	159.2	159.4	159.4	1′′′′	127.8	102.6	102.5	102.5
3	134.8	134.7	134.6	134.6	2′′′′	111.7	72.4	72.3	72.3
4	179.2	179.5	179.5	179.5	3′′′′	149.3	72.3	72.3	72.3
5	163.2	163.0	162.9	162.9	4′′′′	150.6	74.0	74.0	74.7
6	99.8	100.4	100.7	100.7	5′′′′	116.4	69.9	69.9	69.9
7	165.8	163.8	164.4	164.4	6′′′′	124.3	17.6	17.6	17.6
8	94.6	95.7	96.3	96.2	7′′′′	147.5			
9	158.5	157.9	157.8	157.8	8′′′′	115.5			
10	105.8	108.0	107.7	107.7	9′′′′	168.5			
1′	122.7	122.8	122.8	122.8	OCH_3_	56.4			
2′	132.2	132.3	132.2	132.2					
3′	116.3	116.2	116.2	116.9	1′′′′′		105.0	102.2	102.2
4′	161.6	161.6	161.4	161.5	2′′′′′		74.7	72.0	72.0
5′	116.3	116.2	116.2	116.9	3′′′′′		77.8	72.0	72.0
6′	132.2	132.3	132.2	132.2	4′′′′′		71.4	73.8	74.0
					5′′′′′		78.9	69.7	69.7
1′′	100.5	100.2	100.3	100.3	6′′′′′		62.5	17.8	17.8
2′′	74.6	80.0	79.7	79.8					
3′′	84.9	78.9	79.0	79.0	1′′′′′′		127.6	127.6	127.1
4′′	70.0	71.8	72.0	71.7	2′′′′′′		133.4	111.6	131.2
5′′	78.4	78.1	77.2	77.2	3′′′′′′		115.9	149.2	116.2
6′′	62.4	62.6	68.3	68.3	4′′′′′′		160.0	150.5	161.2
					5′′′′′′		115.9	116.5	116.2
1′′′	104.9	99.6	101.6	101.6	6′′′′′′		133.4	124.2	131.2
2′′′	74.8	73.4	74.7	73.8	7′′′′′′		145.0	147.3	147.1
3′′′	77.7	84.3	77.8	77.8	8′′′′′′		116.6	115.1	114.7
4′′′	71.4	69.8	71.7	71.8	9′′′′′′		167.2	169.1	169.1
5′′′	78.1	78.2	75.8	75.7	OCH_3_			56.5	
6′′′	62.5	62.2	64.6	64.6					

**Table 3:** Bioactivities of **7** and **15**–**18** from *C. rotundifolia*.

Isolated Compounds	DPPH (IC_50_, µM)	AGEs (IC_50_, µM)
Kaempferol 3*-O-*[2,6-di*-O-α-*l-rhamnopyranosyl]-β-d-glucoside (**7**)	>100	85.5 ± 3.5
Myricetin 3*-O-*[2,6-di*-O-α-*l-rhamnopyranosyl]-β-d-glucoside (**15**)	43.0 ± 1.15	>100
Myricetin 3*-O-*β-neohesperidoside (**16**)	31.8 ± 0.63	>100
Myricetin 3*-O-*β-d-glucoside (**17**)	14.5 ± 1.15	96.5 ± 1.8
Myricetin (**18**)	11.7 ± 1.8	34.9 ± 1.2
Trolox	29.2 ± 0.39	n.d.
Aminoguanidine hydrochloride	n.d.	7818 ± 34.4

n.d.: not determined.

## Supplementary Materials

The following are available online, Figures S1–S9: HR-ESI-MS, ^1^H, ^13^C NMR, DEPT, COSY, HSQC, HMBC, UV and IR spectra of **1**, Figures S10–S18: Spectra of **2**, Figures S19–S27: Spectra of **3**, Figures S28–S35: Spectra of **4**.

## References

[1] Hall J.C., Sytsma K.J., Iltis H.H. (2002). Phylogeny of Capparaceae and Brassicaceae based on chloroplast sequence data. Am. J. Bot..

[2] Kers L.E., Kubitzki K., Bayer C. (2003). Capparceae. The Families and Genera of Vascular Plants. Flowering Plants, Dicotyledons: Malvales, Capparales, and Non-betalain Caryophyllales..

[3] Migahid A., Hammouda M. (1974). Flora of Saudi Arabia.

[4] Ahmad V., Amber A., Arif S., Chen M.H., Clardy J. (1985). Cadabicine, an alkaloid from *Cadaba farinose*. Phytochemistry.

[5] Al-Musayeib N.M., Mohamed G.A., Ibrahim S.R., Ross S.A. (2013). Lupeol-3-*O*-decanoate, a new triterpene ester from *Cadaba farinose* Frossk. Growing in Saudi Arabia. Med. Chem. Res..

[6] Mohamed G.A., Ibrahim S.R., Al-Musayeib N.M., Ross S.A. (2014). New anti-inflammatory flavonoids from *Cadaba glandulosa* Frossk. Arch. Pharm. Res..

[7] Velmurugan P., Kamaraj M., Prema D. (2010). Phytochemical constituents of *Cadaba trifoliata* Roxb. root extract. Int. J. Phytomed..

[8] Kamel W.M., El-Ghani M.M., El-Bous M.M. (2009). Taxonomic study of Capparaceae from Egypt: Revisited. AJPSB.

[9] Yousif G., Iskander G.M., Eisa E. (1986). Alkaloid components in the Sudan flora. Part II. Alkaloid of *Cadaba farinose* and *C. rotundifolia*. Fitoterapia.

[10] Harbaum B., Hubbermann E.M., Wolf C., Herges R., Zhu Z., Schwarz K. (2007). Identification of flavonoids and hydroxycinnamic acid in pak choi varieties (*Brassica campestris* L. ssp. *chinesis* var. *communis*) by HPLC-ESI-MS^n^ and NMR and their quantification by HPLC-DAD. J. Agric. Food Chem..

[11] Gossan D.P., Alabdul Majed A., Yao-Kouassi P.A., Coffy A.A., Harakat D., Voutaquenne-Nazabadioko L. (2015). New acylated flavonol glycosides from the aerial parts of *Gouania longipetala*. Phytochem. Lett..

[12] Corea G., Fattorusso E., Lanzotti V. (2003). Saponins and flavonoids of *Allium triquetrum*. J. Nat. Prod..

[13] Carotenuto A., Feo V.D., Fattorusso E., Lanzotti V., Magnot S., Cicala C. (1996). The flavonoids of *Allium ursinum*. Phytochemistry.

[14] Séro L., Sanguinet L., Blanchard P., Dang B.T., Morel S., Richomme P., Séraphin D., Séverine D. (2013). Tuning a 96-well microtiter plate fluorescence-based assay to identify AGE inhibitors in crude plant extracts. Molecules.

[15] Sugimoto S., Wanas A.S., Mizuta T., Matsunami K., Kamel M.S., Otsuka H. (2014). Structure elucidation of secondary metabolites isolated from the leaves of *lxora undulate* and their inhibitory activity toward advanced glycation end-products formation. Phytochemistry.

[16] Lee K.T., Choi J.H., Kim D.H., Son K.H., Kim W.B., Kown S.H., Park H.J. (2011). Constituents and antitumor principle of *Allium victorialis* var. *platyphyllum*. Arch. Pharm. Res..

[17] Kazuma K., Noda N., Suzuki M. (2003). Malonylated flavonol glycosides from the petals of *Clitoria ternatea*. Phytochemistry.

[18] Walter A., Sequin U. (1990). Flavonoids from the leaves of *Boscia salicifolia*. Phytochemistry.

[19] Mabry T.J., Markham K.R., Thomas M.B. (1970). The Systematic Identification of Flavonoids.

[20] Zheng X., Li M., Zeng M., Zhang J., Zhao X., Lv J., Zhang Z., Feng W. (2018). Extraction Method of Beitingxinhuangtong C from *Lipidium apetalum* and Its Application in Preparing Estrogenic Drug. Faming Zhuanli Shenging Gongkai Shoumingshu.

[21] Qin X., Xing Y.F., Zhou Z., Yao Y. (2015). Dihydrochalcone compounds isolated from Crabapple leaves showed anticancer effects on human cancer cell line. Molecules.

[22] Yoshikawa M., Sugimoto S., Nakamura S., Matsuda H. (2008). Medicinal flowers. XXII structures of chakasaponins V and VI, chakanoside I, and chakaflavonoside A from flower buds of Chinese tea plant (*Camellia sinensis*). Chem. Pharm. Bull..

[23] Mitani T., Mimura H., Ikeda K., Nishide M., Yamaguchi M., Koyama H., Hayashi Y., Sakamoto H. (2018). Process for the purification of *cis-p*-coumaric acid by cellulose column chromatography after the treatment of the *trans* isomer with ultraviolet irradiation. Anal. Sci..

[24] Matsunami K., Takamori I., Shinzato T., Aramoto M., Kondo K., Otsuka K., Takeda Y. (2006). Radical-scavenging activities of new megastimane glucosides from *Macaranga tanarius* (L.) MULL.-ARG. Chem. Pharm. Bull..

