# Supplementary material for: Four New Flavonoids Isolated from the Aerial Parts of Cadaba rotundifolia Forssk. (Qadab)
Source: Molecules. 2019 Jun 9;24(11):2167. doi: 10.3390/molecules24112167 (PMC6600330; doi:10.3390/molecules24112167)
Supplement: Supplementary file 1 [file molecules-24-02167-s001.pdf]

Supporting information for

Four New Flavonoids Isolated from the Aerial Parts of *Cadaba rotundifolia* Forssk. (Qadab)

Gadah Abdulaziz Al-Hamoud [1,2], Raha Saud Orfali [2,*], Sachiko Sugimoto [1], Yoshi Yamano [1], Nafee Alothyqi [3], Ali Mohammed Alzahrani [4],Katsuyoshi Matsunami [1,*]

[1] Department of Pharmacognosy, Graduate School of Biomedical and Health Sciences, Hiroshima University, 1-2-3 Kasumi, Minami-ku, Hiroshima 734-8553, Japan; matunamil@hiroshima-u.ac.jp (K.M.); yamano@hiroshima-u.ac.jp (Y.Y.); ssugimot@hiroshima-u.ac.jp (S.S.)

[2] Department of Pharmacognosy, College of Pharmacy, King Saud University, Riyadh 11495, Kingdom of Saudi Arabia; galhamoud@ksu.edu.sa (G.A.A.); rorfali@ksu.edu.sa (R.S.O.)

[3] Department of Biology, Umm Al-Qura University, 1109- Makkah Al-Mukarramah, Kingdom of Saudi Arabia; niothyqi@uqu.edu.sa (N.A.)

[4] Department of Biology, Arts and Sciences in Qilwah, 1988- Al-Baha University, alialzahrani@bu.edu.sa (A.M.A.)

* Correspondence: matunamil@hiroshima-u.ac.jp (K.M.); rorfali@ksu.edu.sa (R.S.O.); Tel.: +81-82-257-5335 (K.M.) ; +966-11-8055014 (R.S.O.)

Contents

Figure S1. The HRESIMS spectrum of **1**.
Figure S2. The ^{1}H NMR spectrum of **1** in CD_3OD, 700 MHz.
Figure S3. The ^{13}C NMR spectrum of **1** in CD_3OD, 175 MHz.
Figure S4. The DEPT-135 NMR spectrum of **1** in CD_3OD, 175 MHz.
Figure S5. The COSY spectrum of **1** in CD_3OD, 500 MHz.
Figure S6. The HSQC spectrum of **1** in CD_3OD, 700 MHz.
Figure S7. The HMBC spectrum of **1** in CD_3OD, 700 MHz.
Figure S8. The UV spectrum of **1** in CH_3OH.
Figure S9. The IR spectrum of **1**.
Figure S10. The HRESIMS spectrum of **2**.
Figure S11. The ^{1}H NMR spectrum of **2** in CD_3OD, 600 MHz.
Figure S12. The ^{13}C NMR spectrum of **2** in CD_3OD, 150 MHz.
Figure S13. The DEPT-135 NMR spectrum of **2** in CD_3OD, 150 MHz.
Figure S14. The COSY spectrum of **2** in CD_3OD, 600 MHz.
Figure S15. The HSQC spectrum of **2** in CD_3OD, 600 MHz.
Figure S16. The HMBC spectrum of **2** in CD_3OD, 600 MHz.
Figure S17. The UV spectrum of **2** in CH_3OH.
Figure S18. The IR spectrum of **2**.
Figure S19. The HRESIMS spectrum of **3**.
Figure S20. The ^{1}H NMR spectrum of **3** in CD_3OD, 600 MHz.
Figure S21. The ^{13}C NMR spectrum of **3** in CD_3OD, 150 MHz.
Figure S22. The DEPT-135 NMR spectrum of **3** in CD_3OD, 125 MHz.
Figure S23. The COSY spectrum of **3** in CD_3OD, 600 MHz.
Figure S24. The HSQC spectrum of **3** in CD_3OD, 600 MHz.
Figure S25. The HMBC spectrum of **3** in CD_3OD, 600 MHz.
Figure S26. The UV spectrum of **3** in CH_3OH.
Figure S27. The IR spectrum of **3**.
Figure S28. The HRESIMS spectrum of **4**.
Figure S29. The ^{1}H NMR spectrum of **4** in CD_3OD, 600 MHz
Figure S30. The ^{13}C NMR spectrum of **4** in CD_3OD, 150 MHz.
Figure S31. The COSY spectrum of **4** in CD_3OD, 600 MHz
Figure S32. The HSQC spectrum of **4** in CD_3OD, 600 MHz
Figure S33. The HMBC spectrum of **4** in CD_3OD, 600 MHz
Figure S34. The UV spectrum of **4** in CH_3OH.
Figure S35. The IR spectrum of **4**.

Figure S1. The HRESIMS spectrum of **1**.

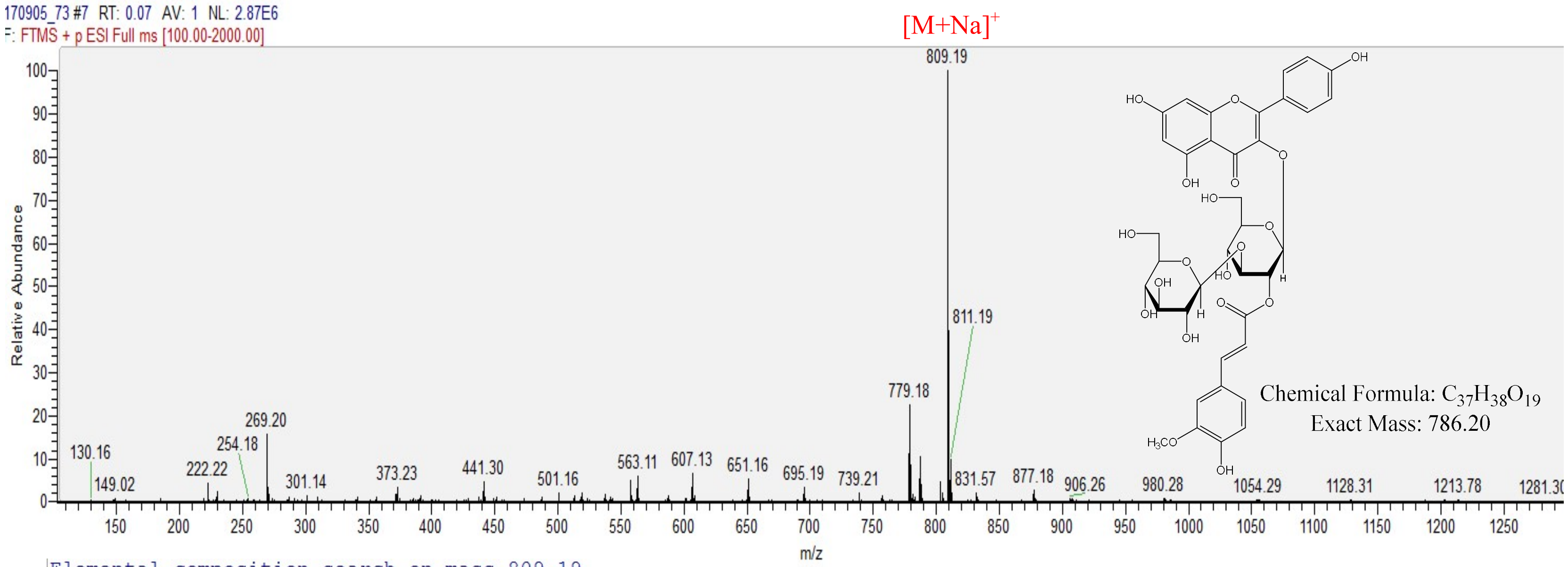

Elemental composition search on mass 809.19

m/z= 804.19-814.19

m/z	Theo. Mass	Delta (mmu)	RDB equiv.	Composition
809.1888	809.1899	-1.11	18.5	C 37 H 38 O 19 Na

Figure S2. The ^{1}H NMR spectrum of **1** in CD_3OD, 700 MHz.

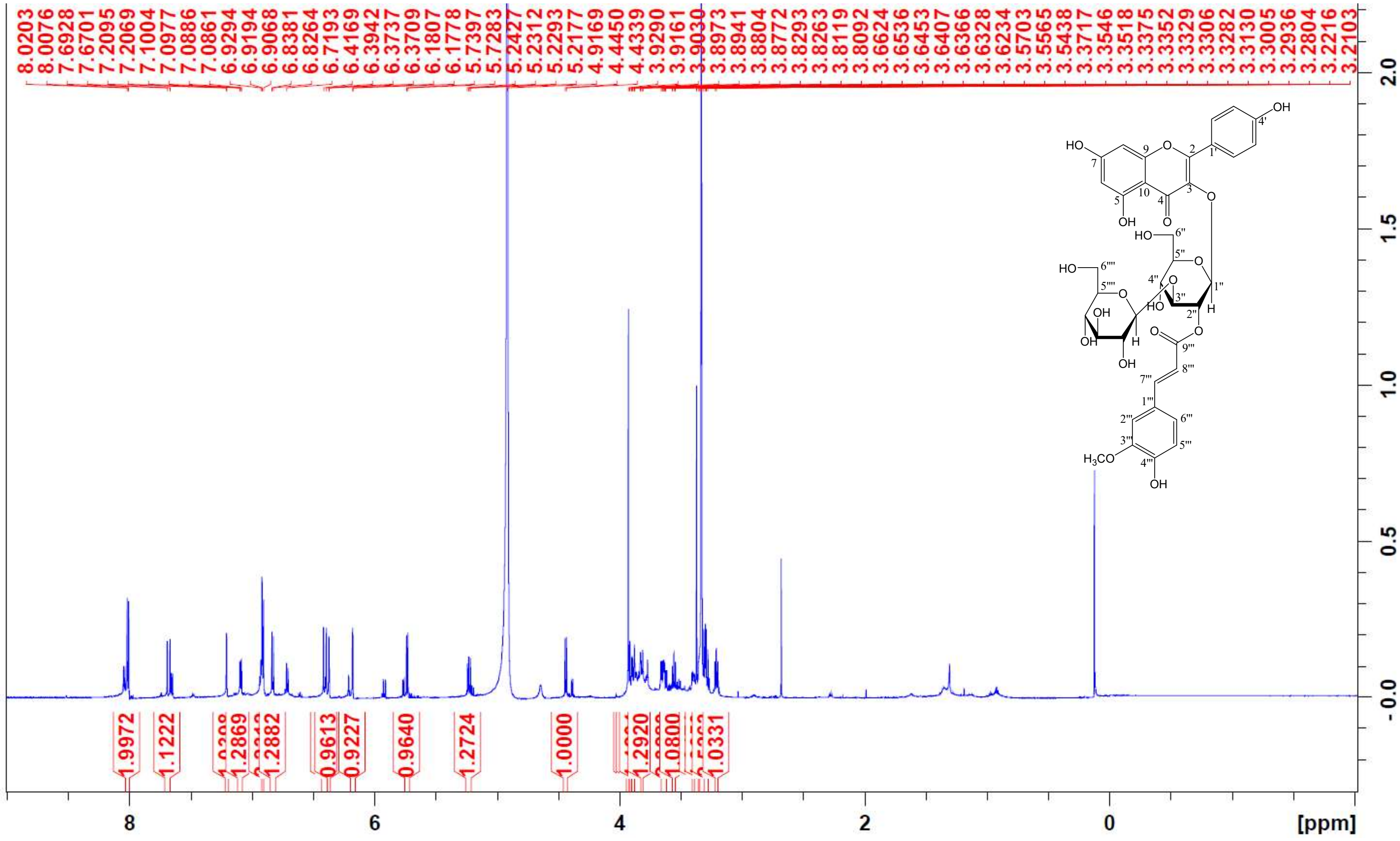

Figure S3. The ^{13}C NMR spectrum of **1** in CD_3OD, 175 MHz.

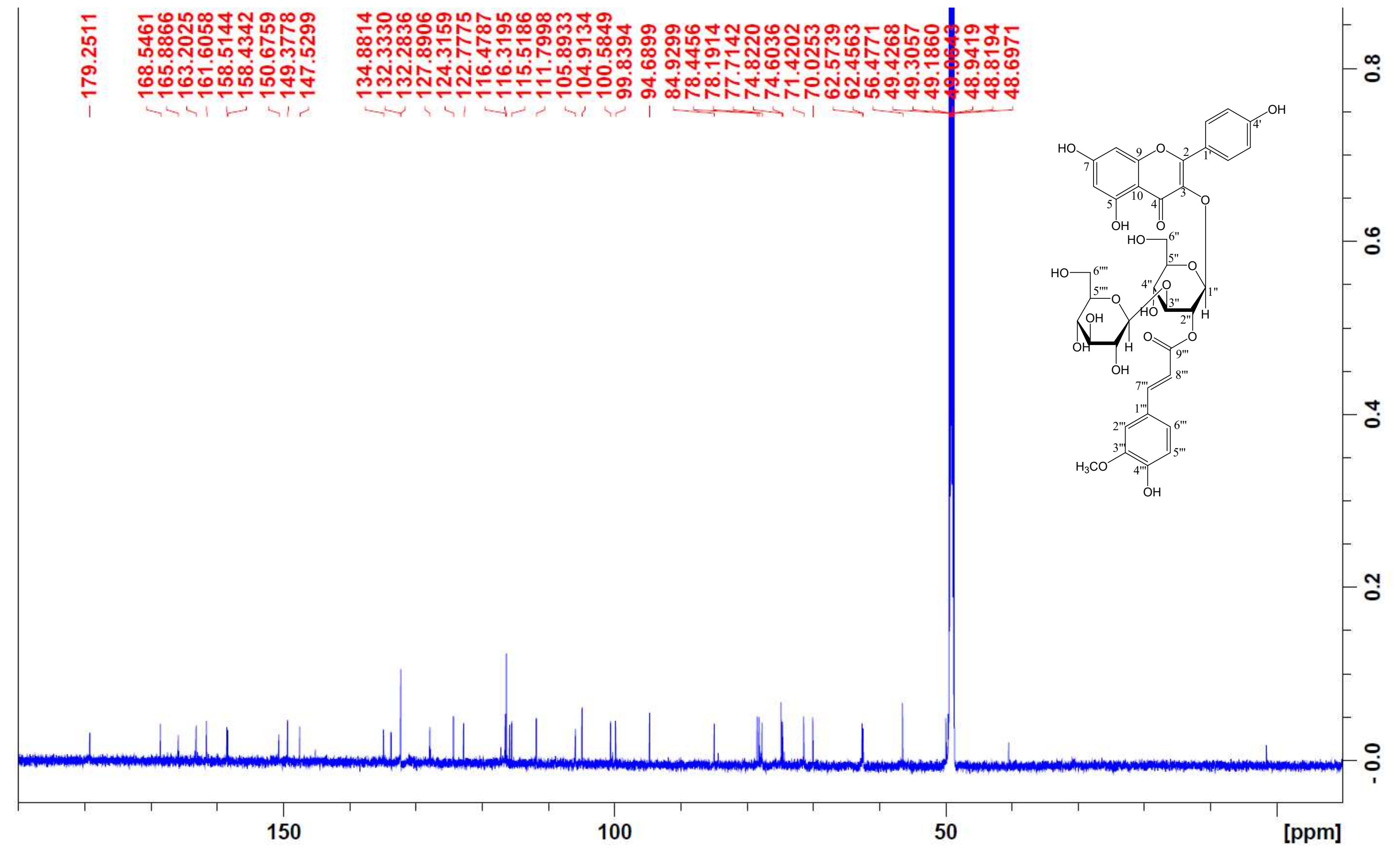

Figure S4. The DEPT-135 NMR spectrum of **1** in CD_3OD, 175 MHz.

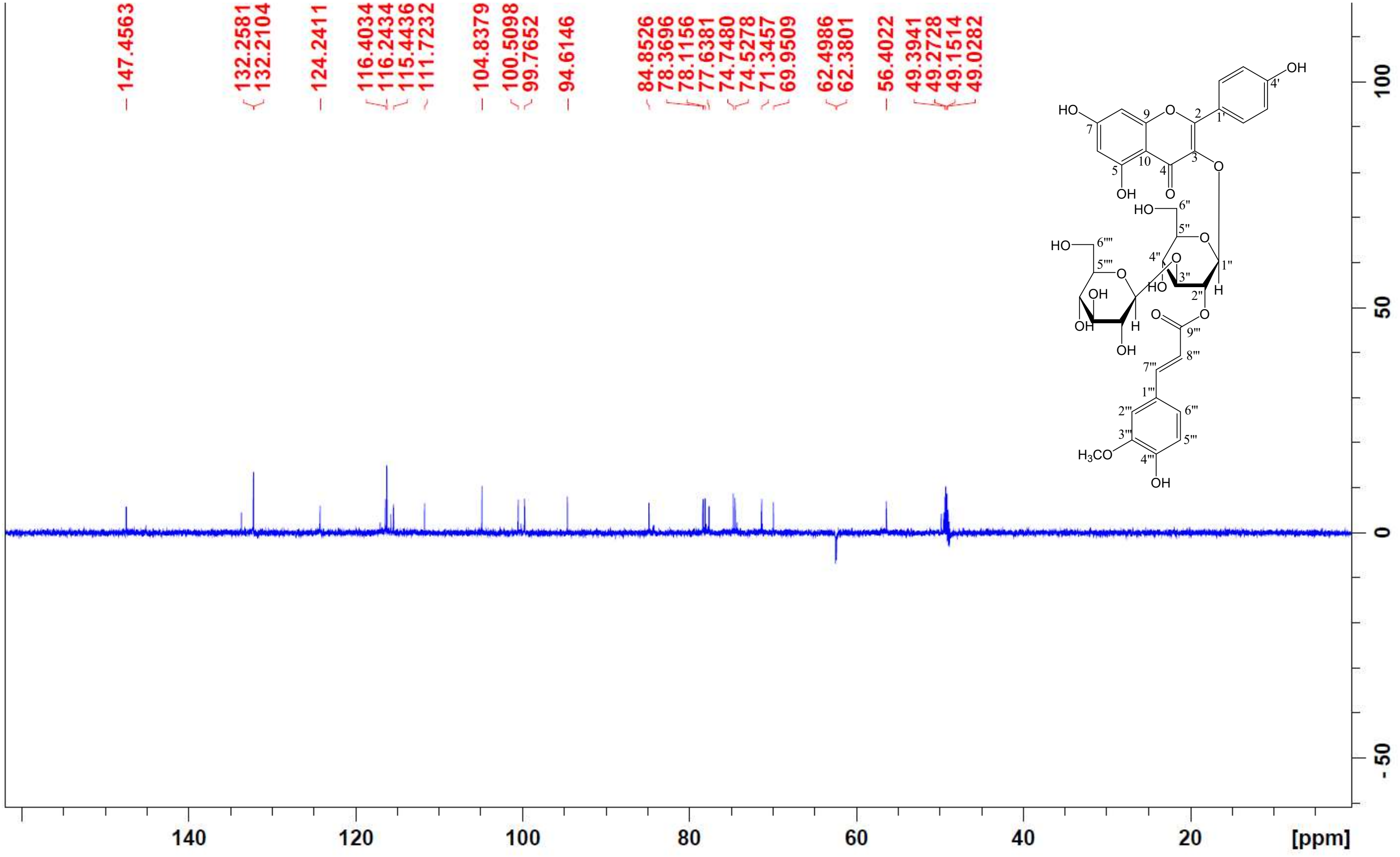

Figure S5. The COSY spectrum of **1** in CD_3OD, 500 MHz.

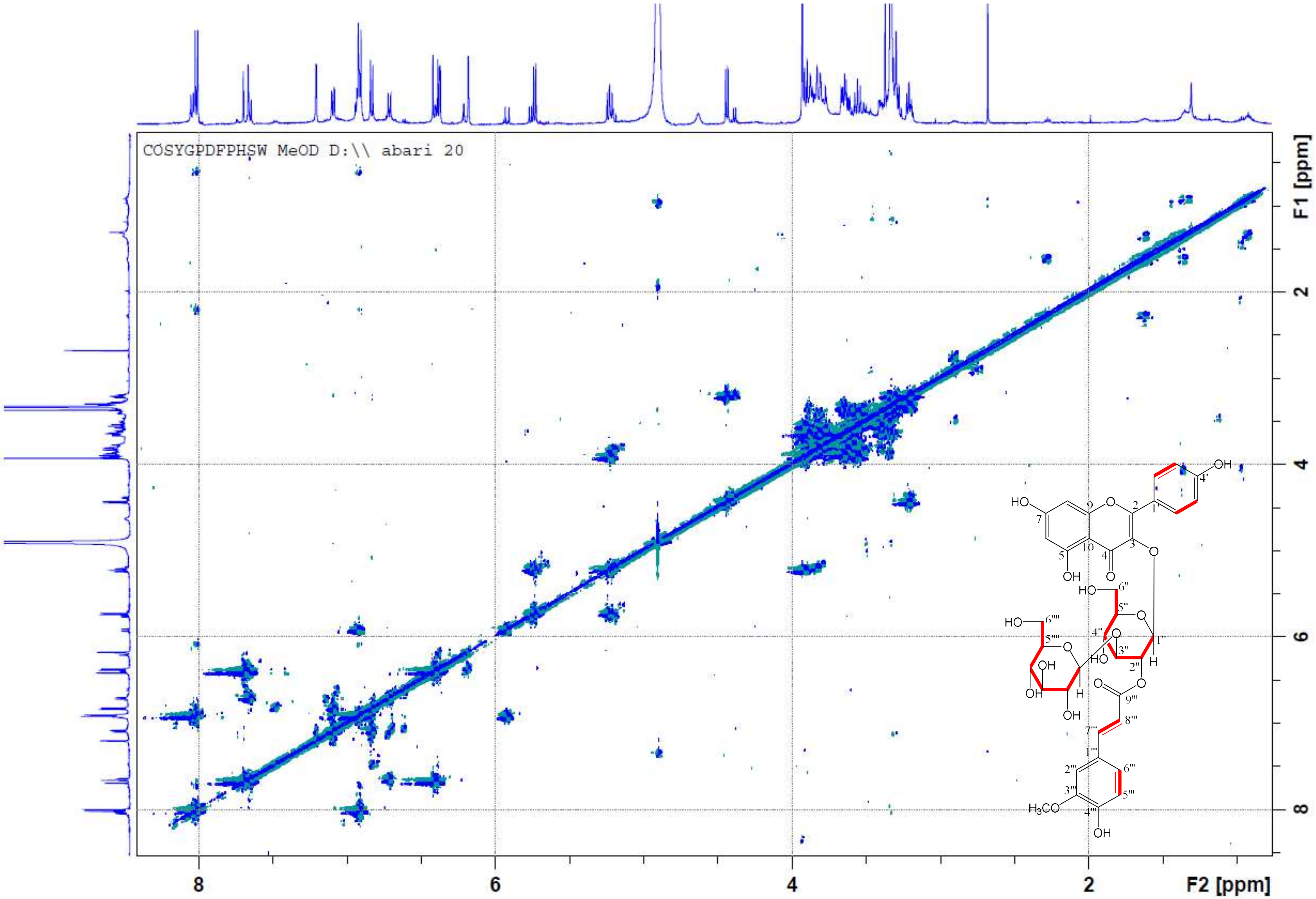

Figure S6. The HSQC spectrum of **1** in CD_3OD, 700 MHz.

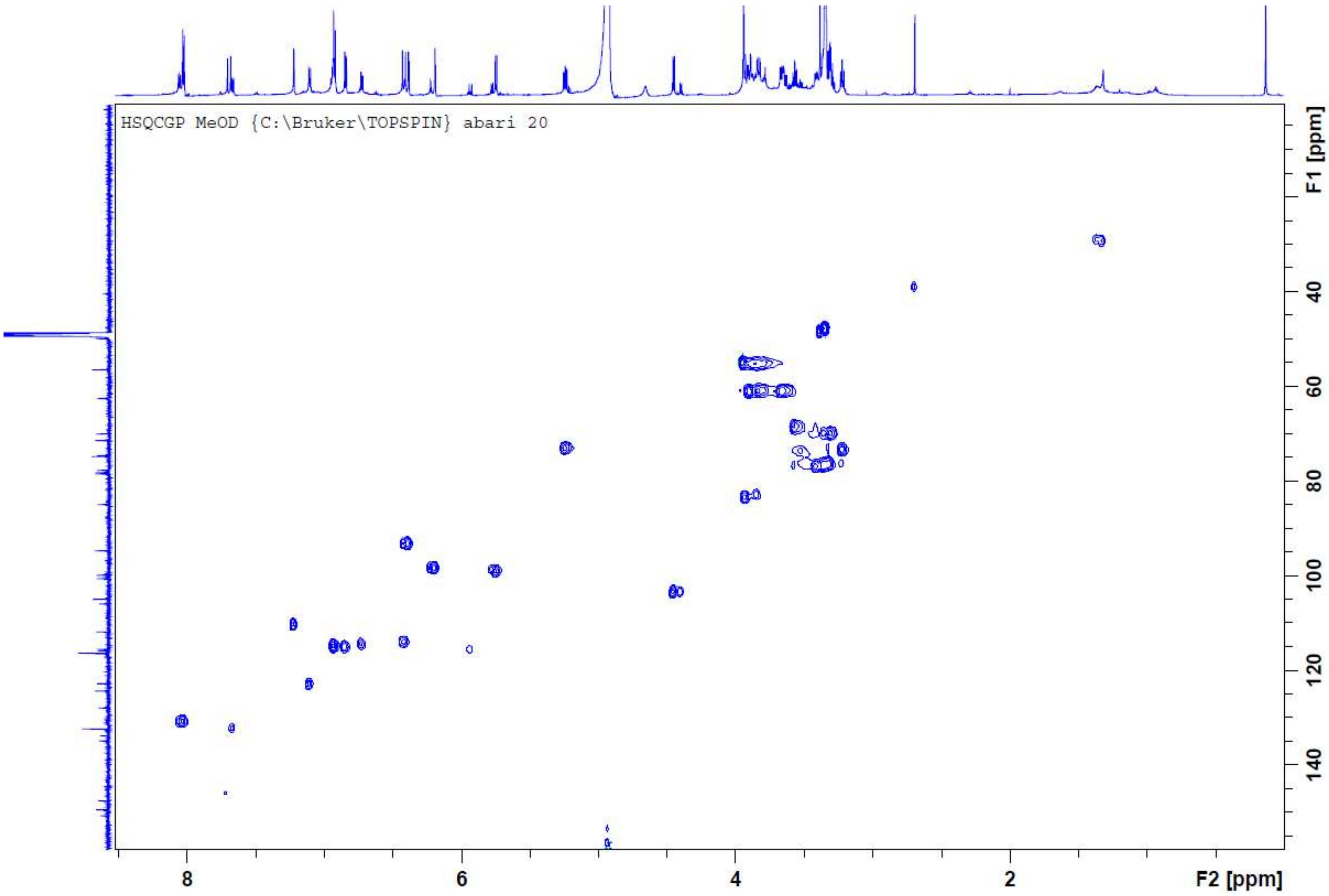

Figure S7. The HMBC spectrum of **1** in CD_3OD, 700 MHz

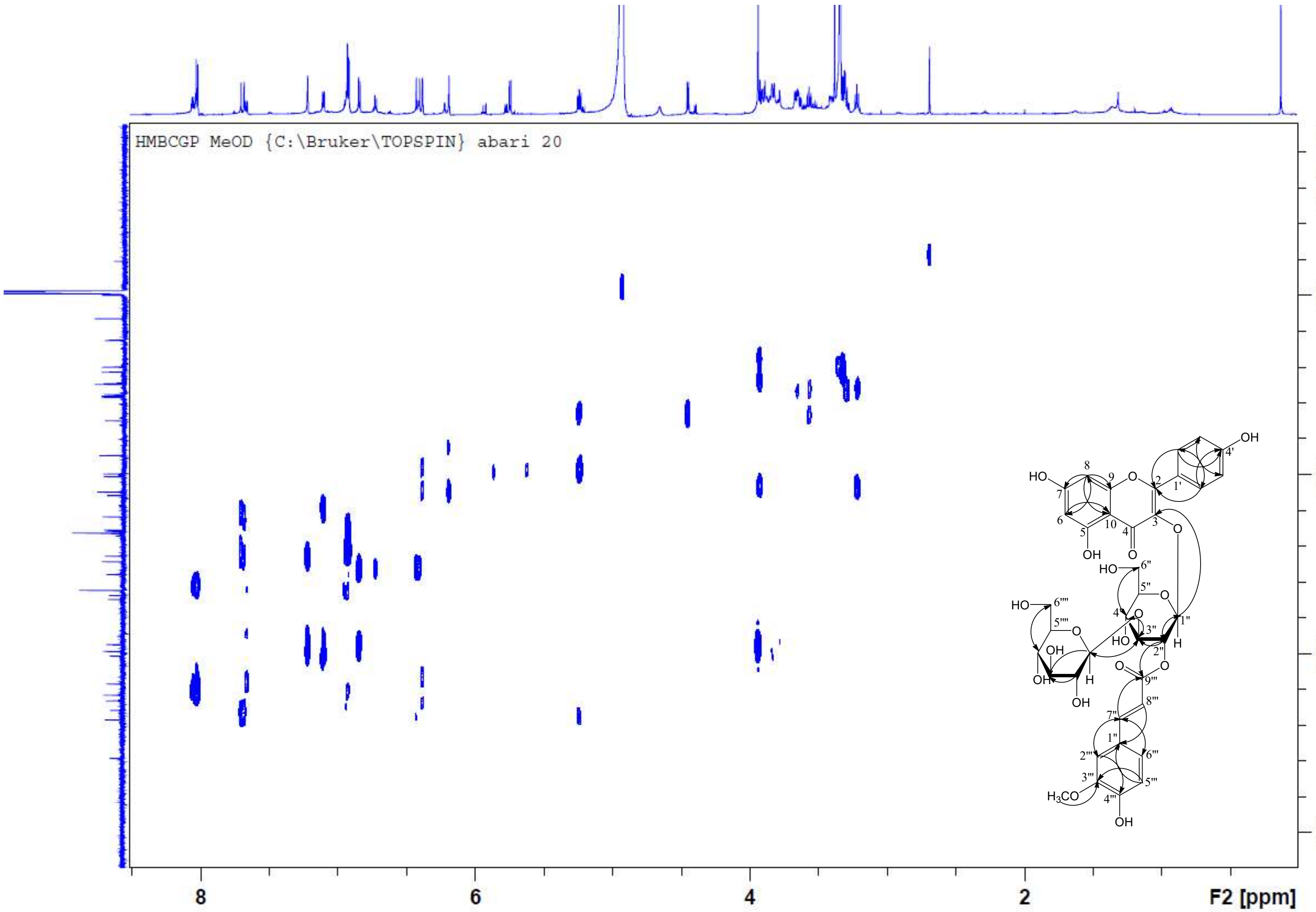

Figure S8. The UV spectrum of **1** in CH_3OH.

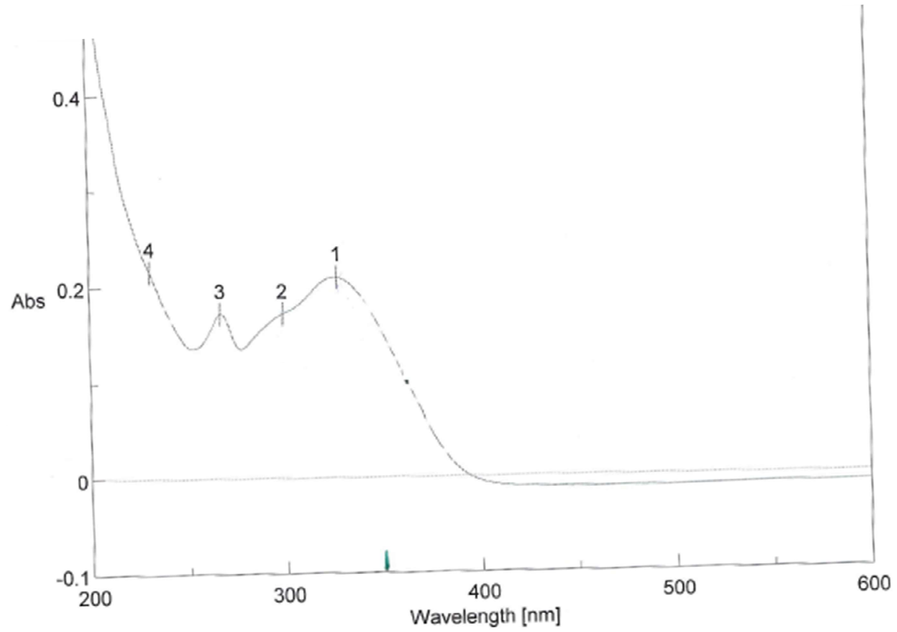

[コメント情報]
試料名
コメント
測定者
所属
会社 HU

[データ情報]	
作成日時	2018/07/02 20:18
データタイプ	等間隔データ
横軸	Wavelength [nm]
縦軸	Abs
スタート	600 nm
エンド	200 nm
データ間隔	1 nm
データ数	401

[測定情報]	
装置名	V-630
機種名	V-630
シリアル番号	A042161148
測定日時	2018/07/02 20:18
測光モード	Abs
測定範囲	600 - 200 nm
データ取込間隔	1 nm
UV/Vis バンド幅	1.5 nm
レスポンス	Fast
走査速度	400 nm/min
光源切換	340 nm
光源	D2/WI
フィルタ切換	ステップ
補正	ベースライン

[ピーク検出結果]

No.	位置	強度	No.	位置	強度
1	327	0.208647	2	299	0.170714
3	267	0.171392	4	231	0.215791

Figure S9. The IR spectrum of **1**.

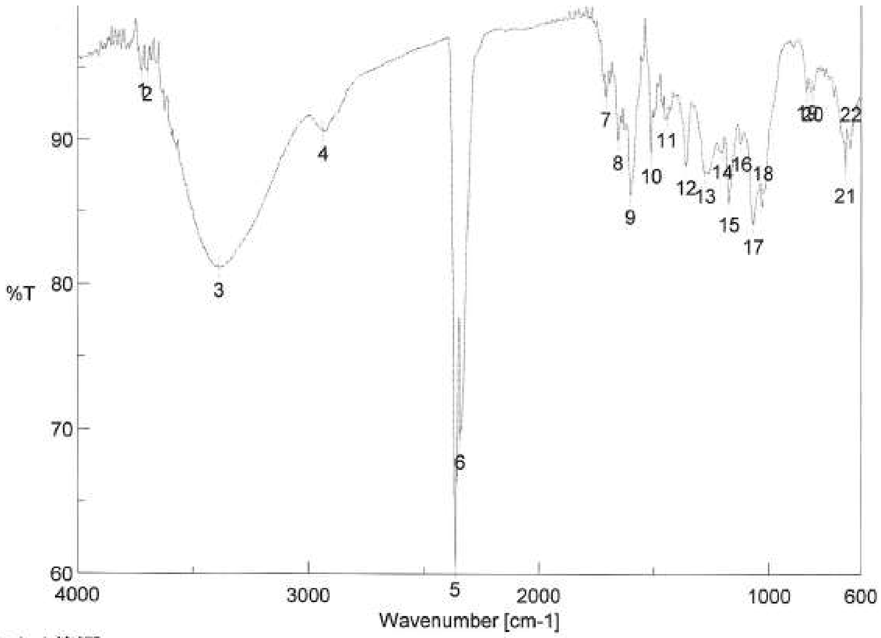

[コメント情報]

試料名	
コメント	
測定者	
所属	
会社	HU

[データ情報]		[測定情報]	
作成日時	2018/06/28 17:13	機種名	FT/IR-4600typeA
		シリアル番号	D015461786
データタイプ	等間隔データ		
横軸	Wavenumber [cm-1]	測定日時	2018/06/28 17:13
縦軸	%T		
スタート	599.753 cm-1	光源	標準光源
エンド	7800.65 cm-1	検出器	TGS
データ間隔	0.964233 cm-1	積算回数	10
データ数	7469	分解	4 cm-1
		ゼロフィリング	On
		アポダイゼーション	Cosine
		ゲイン	Auto (2)
		アパーチャー	Auto (7.1 mm)
		スキャンスピード	Auto (2 mm/sec)
		フィルタ	Auto (30000 Hz)

[ピーク検出結果]

No.	位置	強度	No.	位置	強度
1	3721.94	94.9495	2	3698.8	94.7024
3	3388.32	81.1179	4	2936.09	90.5848
5	2363.34	60.4968	6	2341.16	69.2916
7	1710.55	92.9836	8	1658.48	89.9664
9	1604.48	86.2189	10	1513.85	89.0788
11	1446.35	91.5601	12	1363.43	88.2767
13	1280.5	87.7144	14	1205.29	89.38

[ピーク検出結果]

No.	位置	強度	No.	位置	強度
15	1176.36	85.7295	16	1122.37	89.9077
17	1070.3	84.2004	18	1028.84	86.1398
19	836.955	93.4566	20	810.92	93.3788
21	669.178	87.7475	22	645.072	90.2335

Figure S10. The HRESIMS spectrum of **2**.

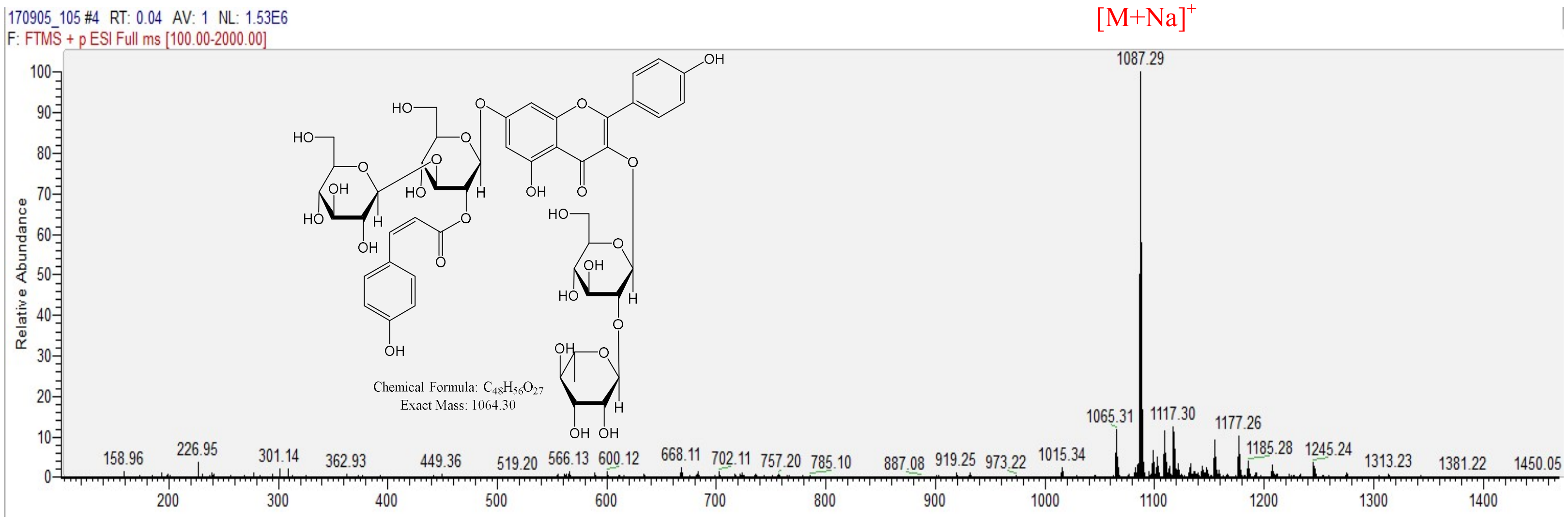

Elemental composition search on mass 1087.29

m/z= 1082.29-1092.29

m/z	Theo. Mass	Delta (mmu)	RDB equiv.	Composition
1087.2875	1087.2901	-2.64	20.5	$C_{48}H_{56}O_{27}Na$

Figure S11. The ^{1}H NMR spectrum of **2** in CD_3OD, 600 MHz.

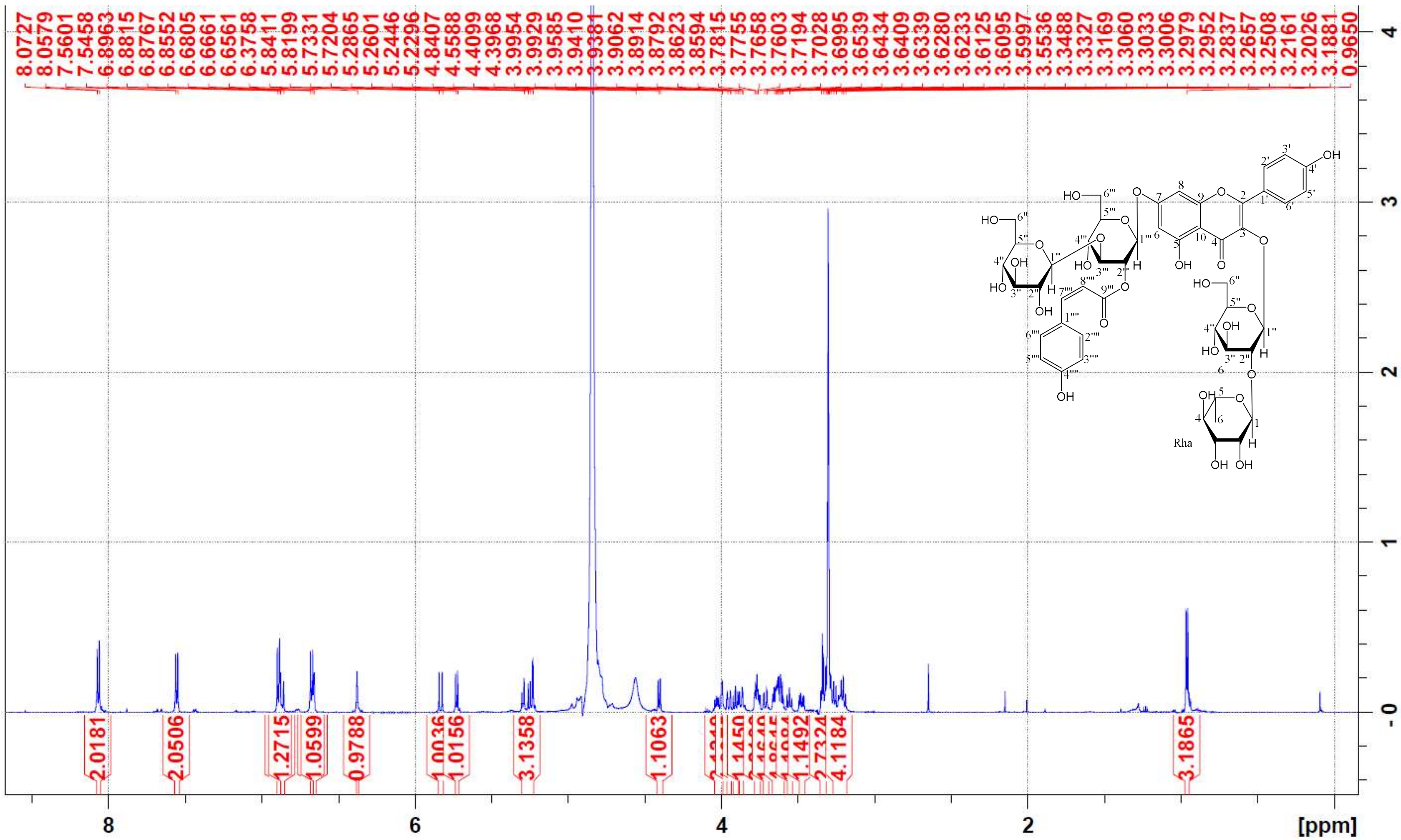

Figure S12. The ^{13}C NMR spectrum of **2** in CD_3OD, 150 MHz.

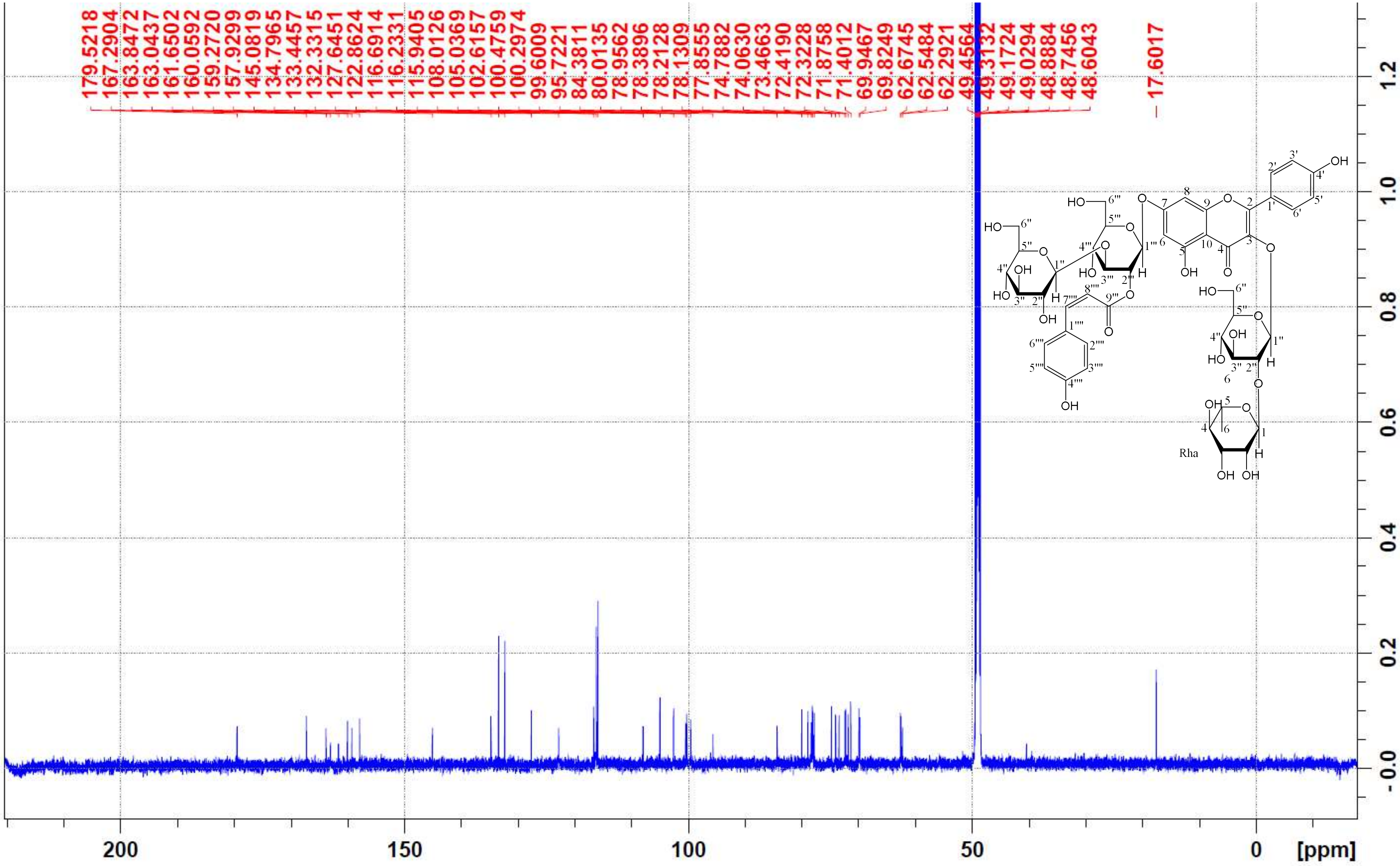

Figure S13. The DEPT-135 spectrum of **2** in CD_3OD, 150 MHz.

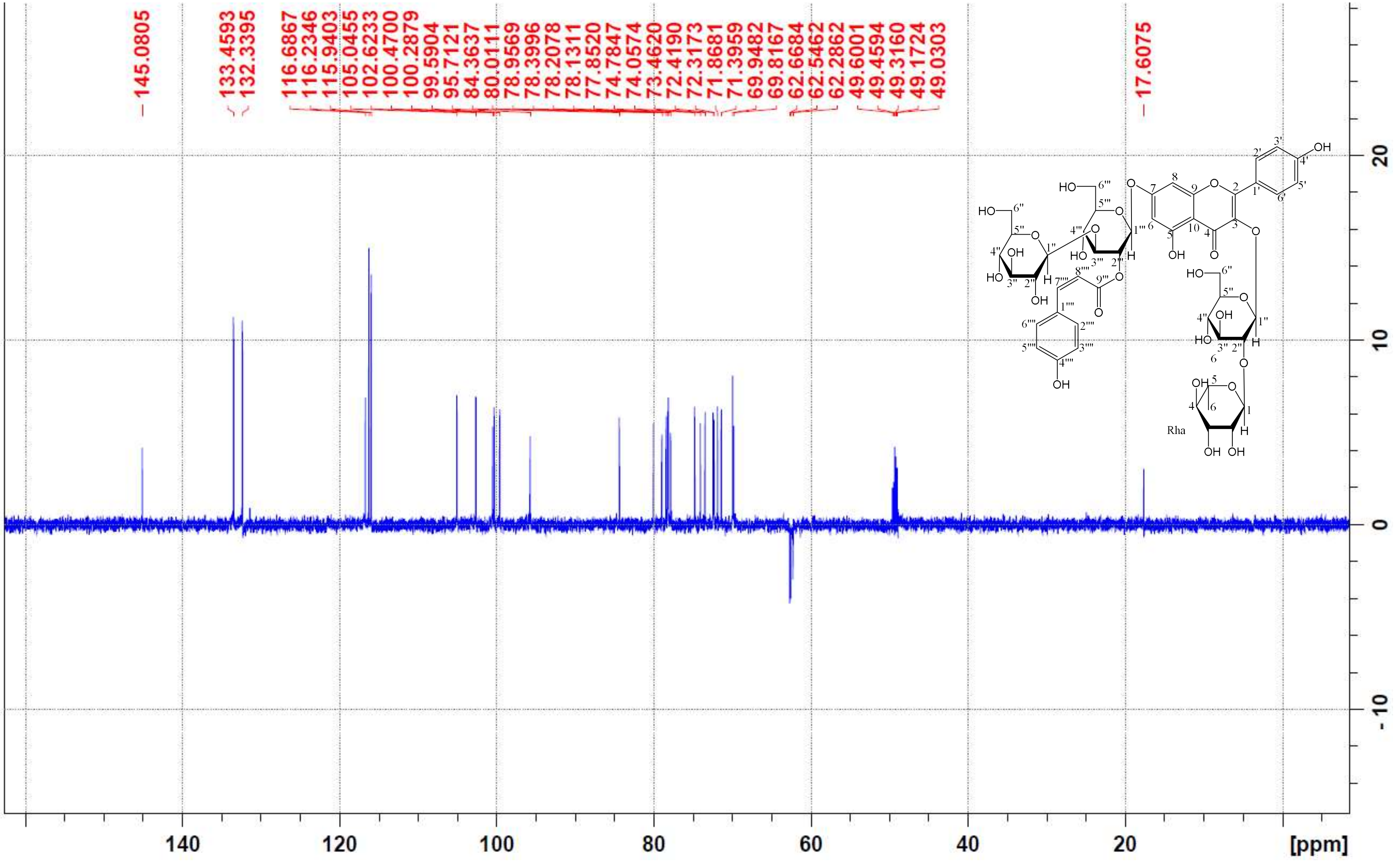

Figure S14. The COSY spectrum of **2** in CD_3OD, 600 MHz.

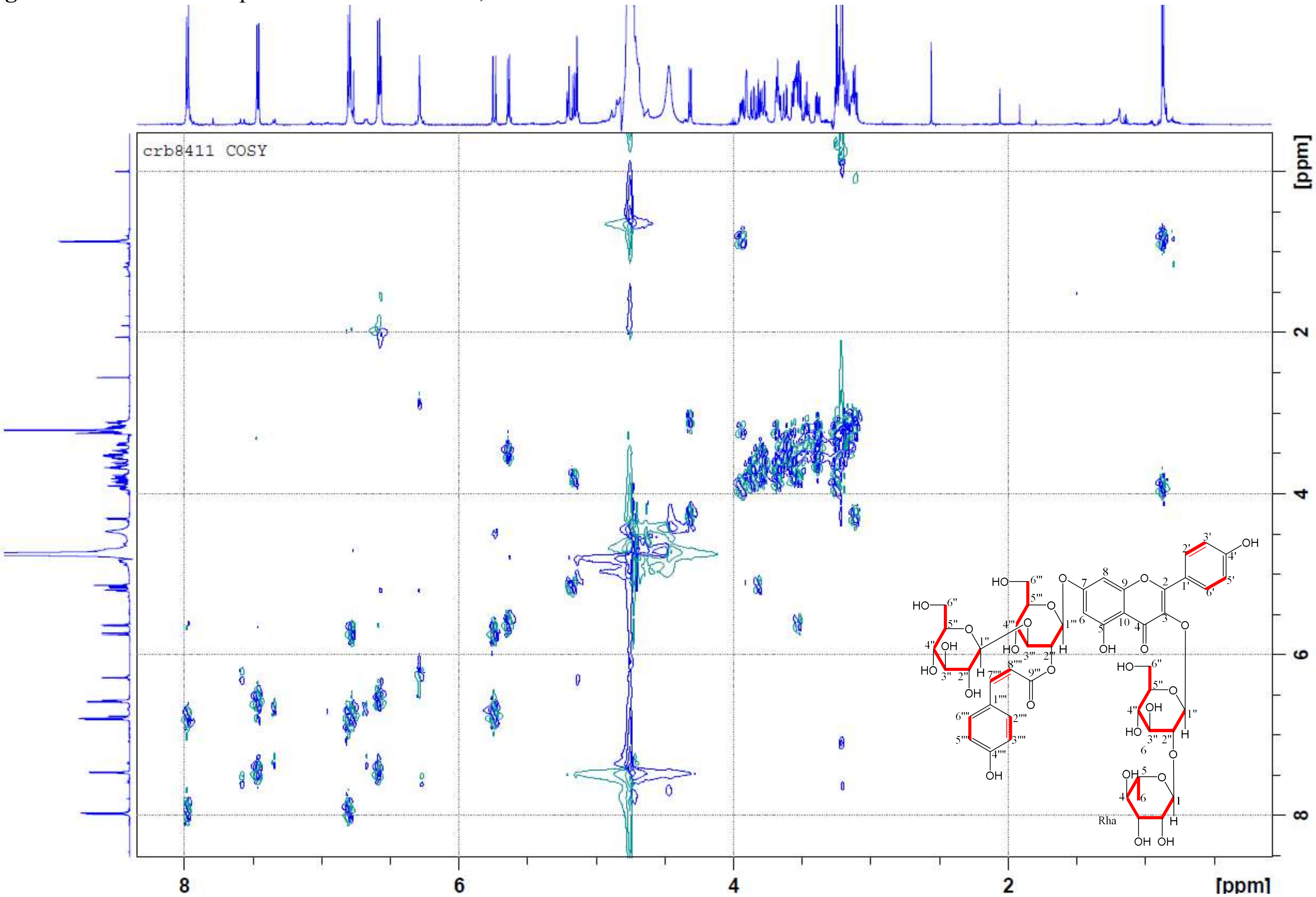

Figure S15. The HSQC spectrum of **2** in CD_3OD, 600 MHz.

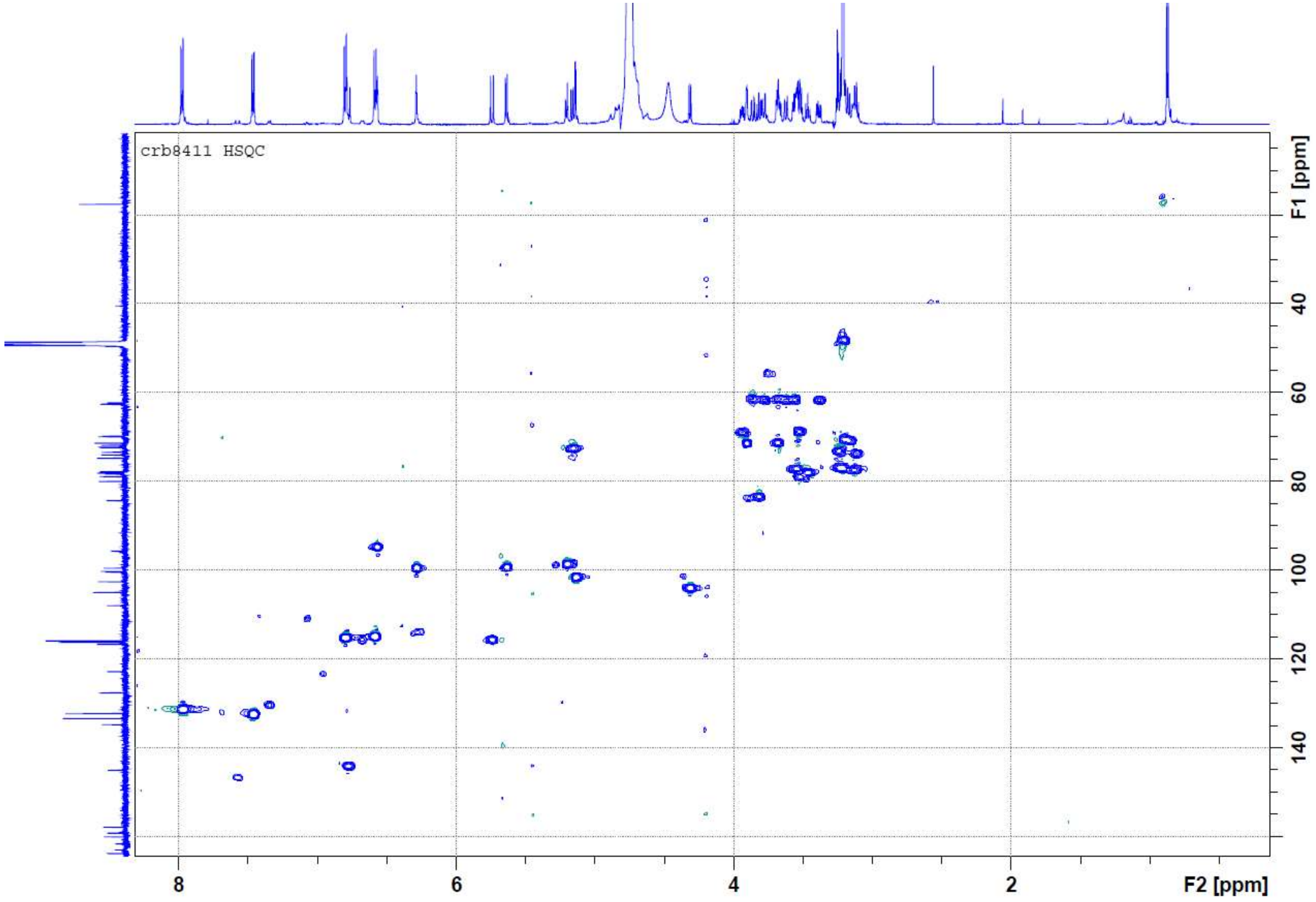

Figure S16. The HMBC spectrum of **2** in CD_3OD, 600 MHz.

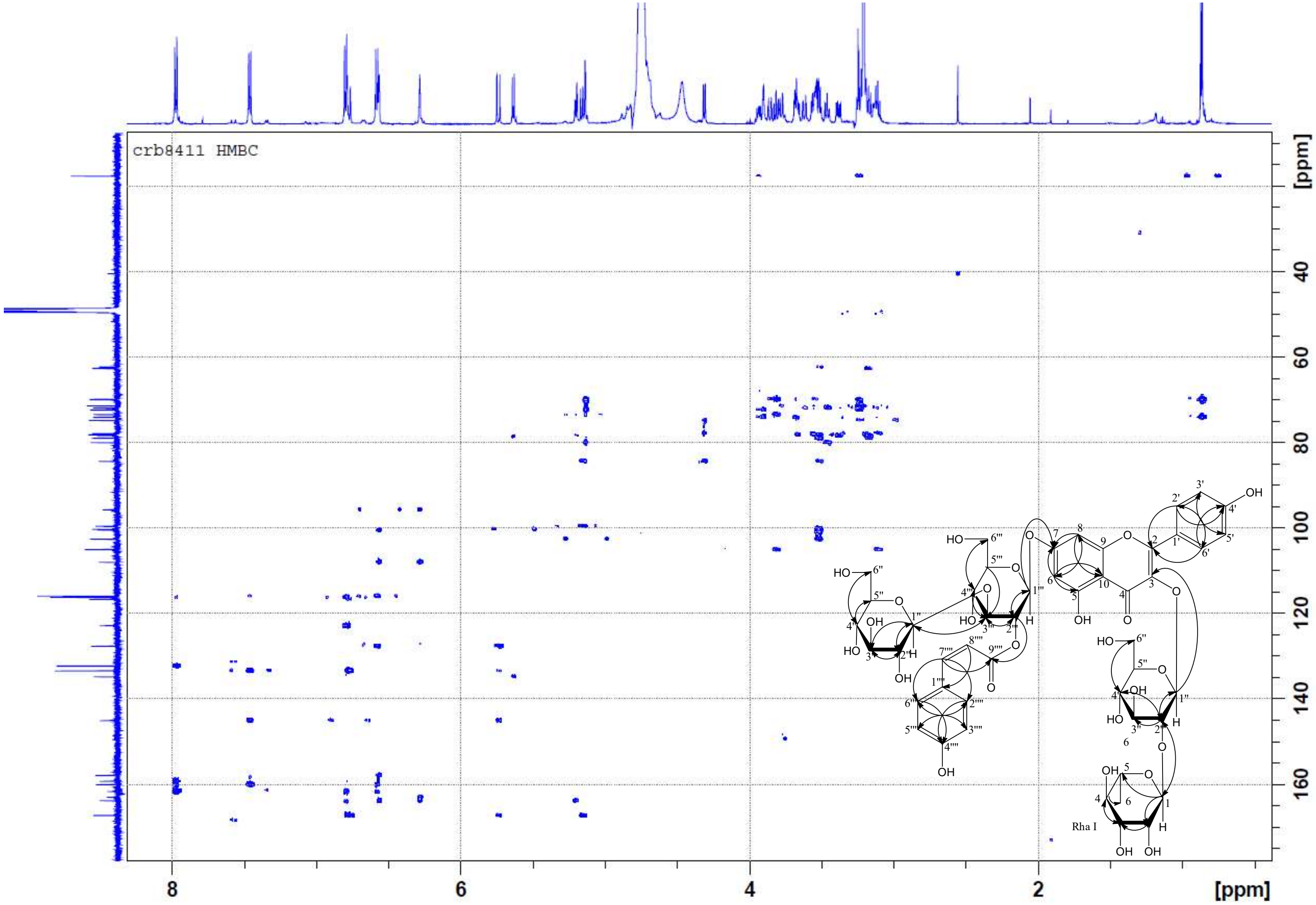

Figure S17. The UV spectrum of **2** in CH_3OH.

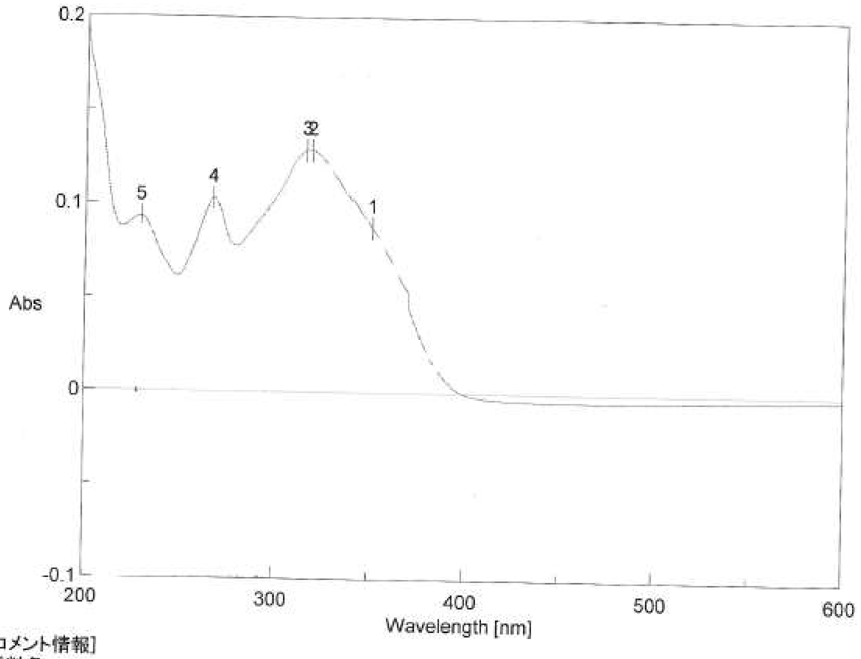

[コメント情報]
試料名
コメント
測定者
所属
会社　HU

[データ情報]
作成日時　2018/07/04 18:32
データタイプ　等間隔データ
横軸　Wavelength [nm]
縦軸　Abs
スタート　600 nm
エンド　200 nm
データ間隔　1 nm
データ数　401

[測定情報]
装置名　V-630
機種名　V-630
シリアル番号　A042161148
測定日時　2018/07/04 18:31
測光モード　Abs
測定範囲　600 - 200 nm
データ取込間隔　1 nm
UV/Vis バンド幅　1.5 nm
レスポンス　Fast
走査速度　400 nm/min
光源切換　340 nm
光源　D2/WI
フィルタ切換　ステップ
補正　ベースライン

[ピーク検出結果]

No.	位置	強度	No.	位置	強度
1	351	0.0878893	2	319	0.128869
3	316	0.128939	4	267	0.102983
5	229	0.0929446		= 305	0.98a

Figure S18. The IR spectrum of **2**.

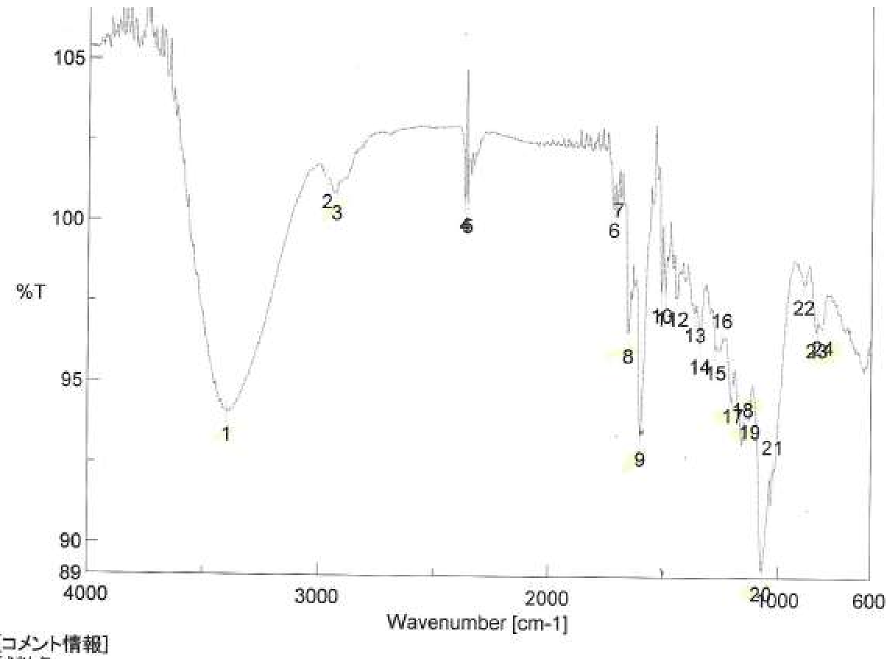

[コメント情報]

試料名	
コメント	
測定者	
所属	
会社	HU

[データ情報]

作成日時	2018/07/04 15:56
データタイプ	等間隔データ
横軸	Wavenumber [cm-1]
縦軸	%T
スタート	599.753 cm-1
エンド	7800.65 cm-1
データ間隔	0.964233 cm-1
データ数	7469

[測定情報]

機種名	FT/IR-4600typeA
シリアル番号	D015461786
測定日時	2018/07/04 15:56
光源	標準光源
検出器	TGS
積算回数	10
分解	4 cm-1
ゼロフィリング	On
アポダイゼーション	Cosine
ゲイン	Auto (2)
アパーチャー	Auto (7.1 mm)
スキャンスピード	Auto (2 mm/sec)
フィルタ	Auto (30000 Hz)

[ピーク検出結果]

No.	位置	強度	No.	位置	強度
1	3397.96	94.0493	2	2965.98	101.287
3	2924.52	100.947	4	2367.19	100.627
5	2357.55	100.577	6	1721.16	100.471
7	1701.87	101.107	8	1657.52	96.5757
9	1603.52	93.3858	10	1512.88	97.8359
11	1493.6	97.7406	12	1442.49	97.7332
13	1367.28	97.2424	14	1345.11	96.2604

[ピーク検出結果]

No.	位置	強度	No.	位置	強度
15	1276.65	96.0813	16	1250.61	96.2852
17	1201.43	94.748	18	1157.08	93.5446
19	1126.22	94.2661	20	1072.23	89.2203
21	1024.98	92.3861	22	893.844	98.1213
23	836.955	96.7832	24	810.92	96.8669

Figure S19. The HRESIMS spectrum of **3**.

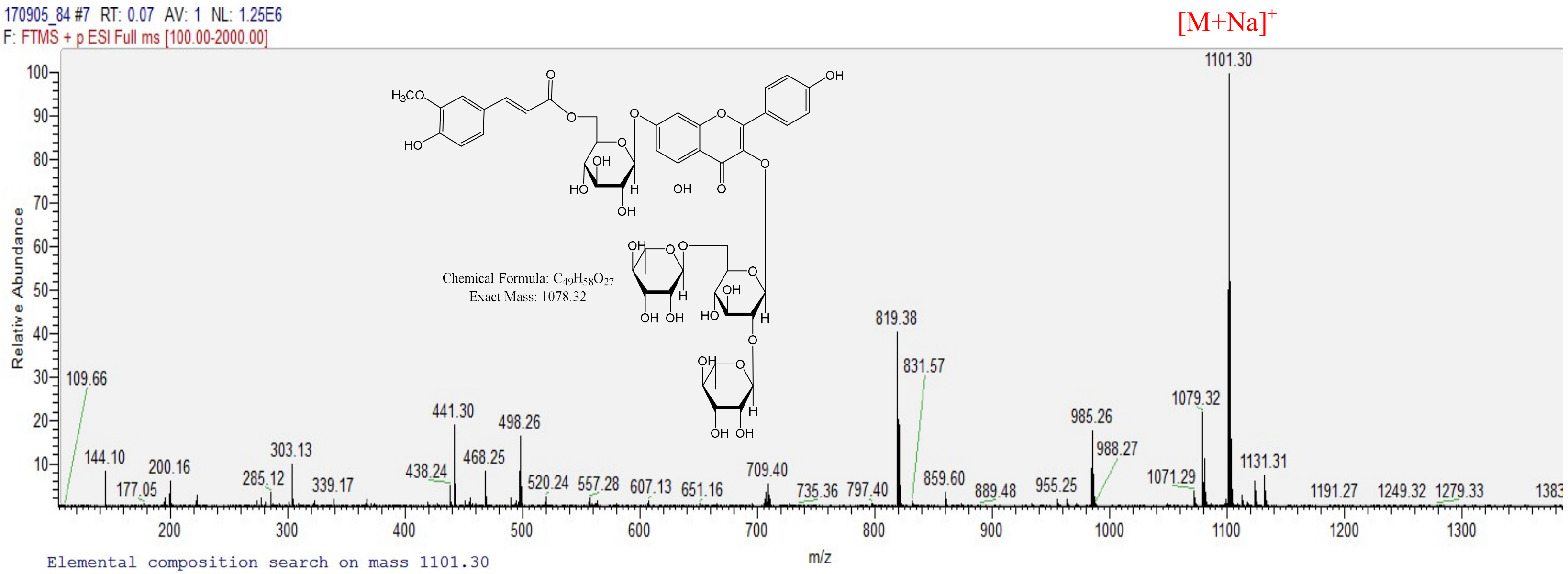

Elemental composition search on mass 1101.30

m/z= 1096.30-1106.30

m/z	Theo. Mass	Delta (mmu)	RDB equiv.	Composition
1101.3021	1101.3058	-3.65	20.5	$C_{49}H_{58}O_{27}Na$

Figure S20. The ^{1}H NMR spectrum of **3** in CD_3OD, 600 MHz.

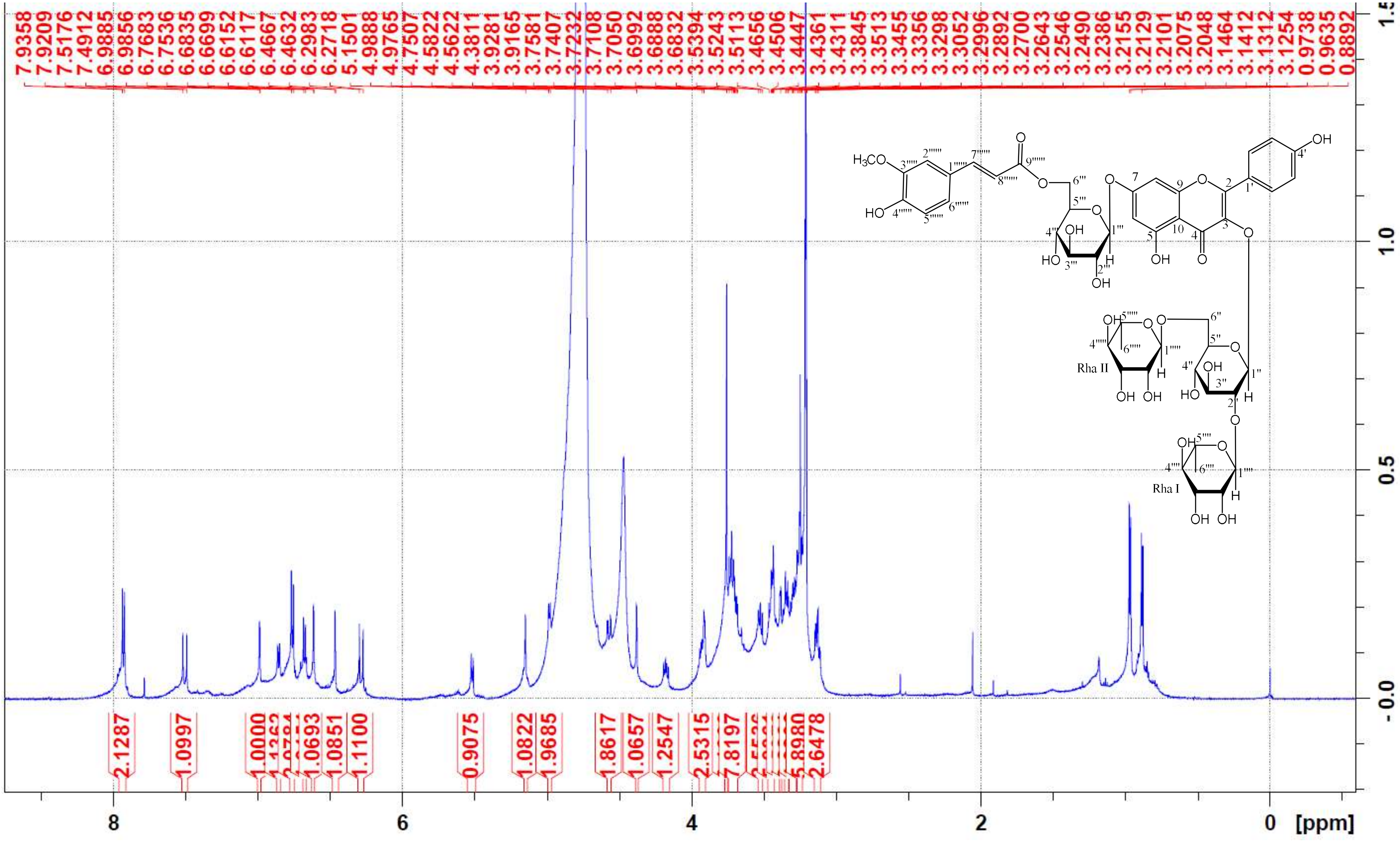

Figure S21. The ^{13}C NMR spectrum of **3** in CD_3OD, 150 MHz.

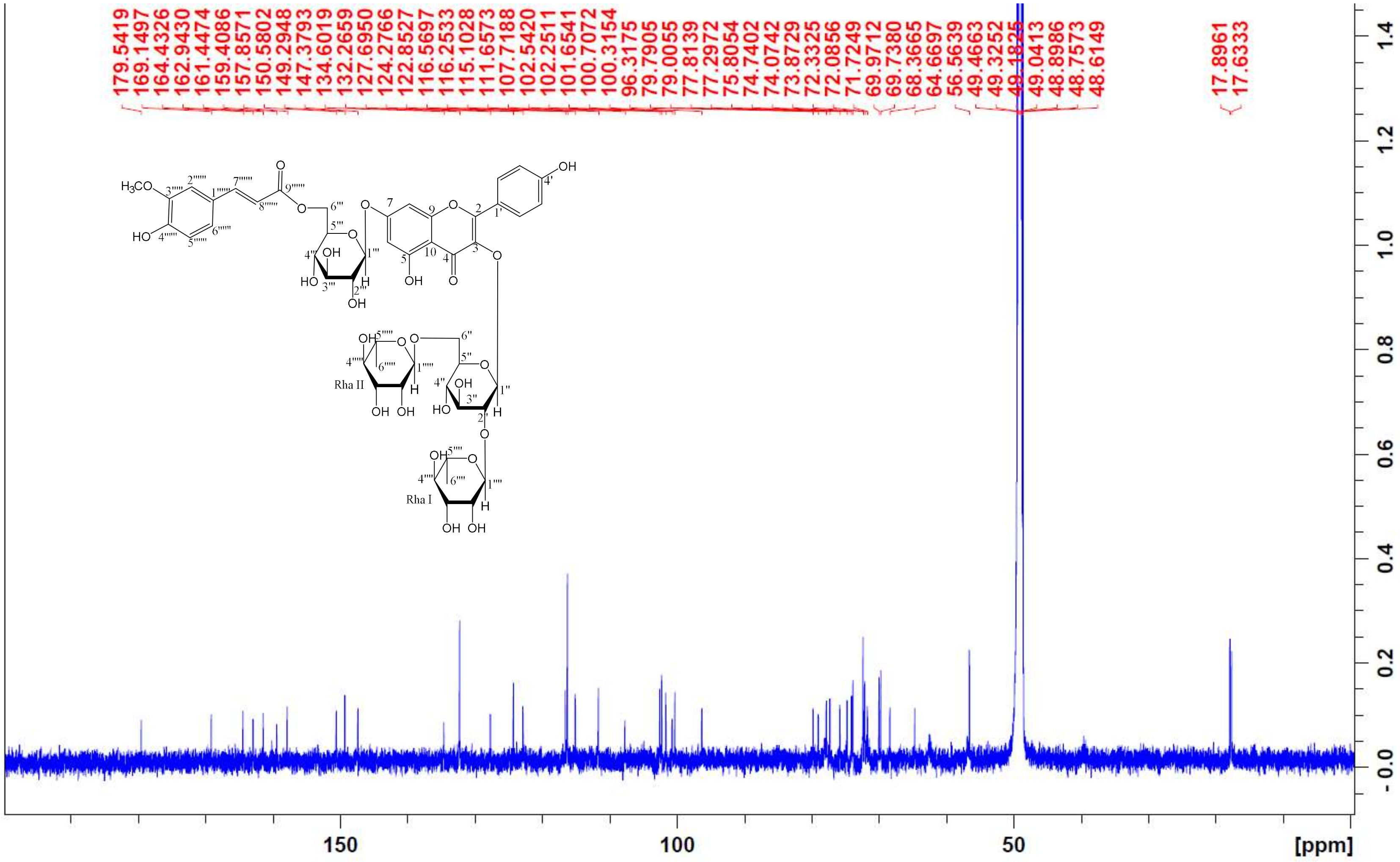

Figure S22. The DEPT-135 spectrum of **3** in CD_3OD, 150 MHz.

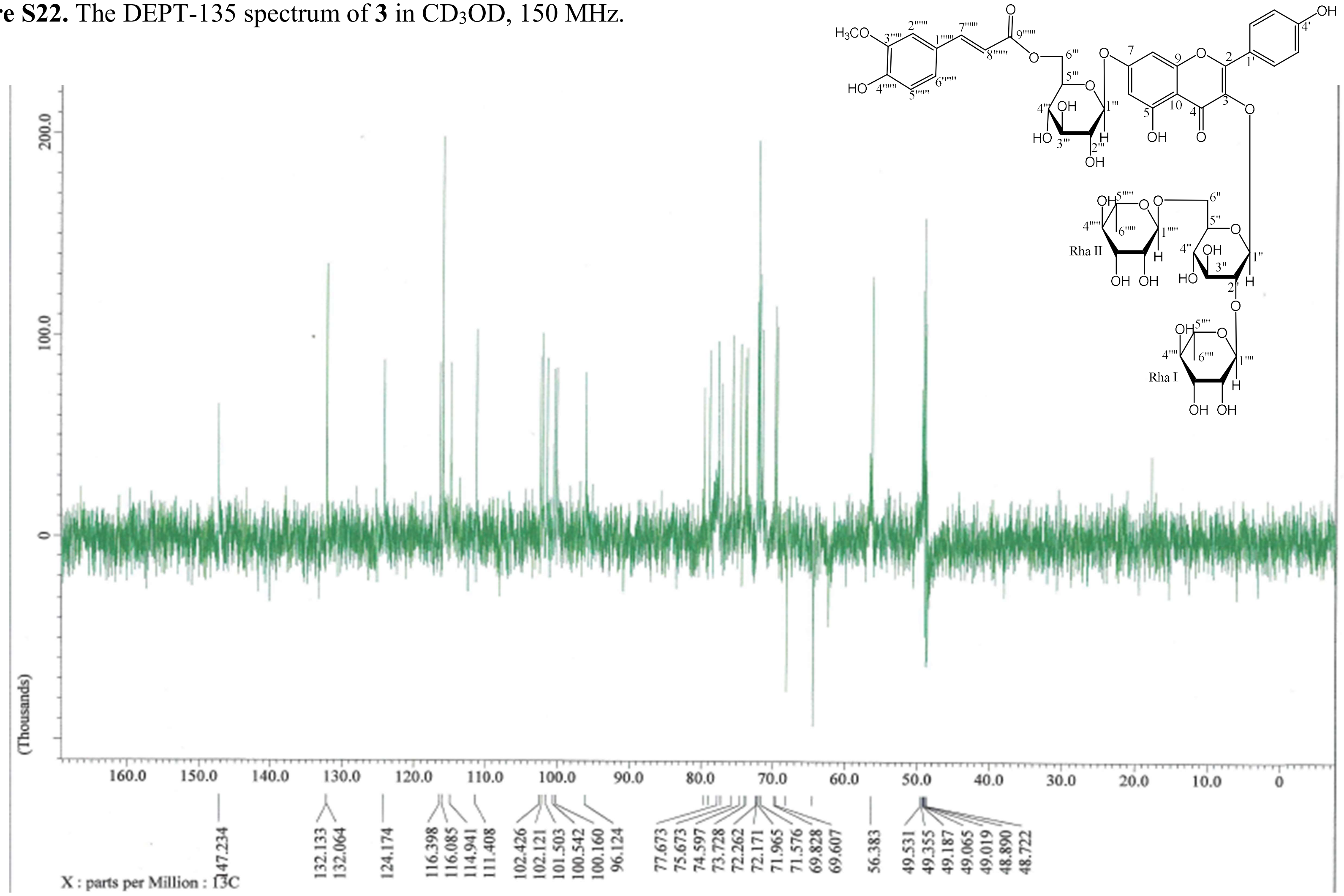

Figure S23. The COSY spectrum of **3** in CD_3OD, 600 MHz.

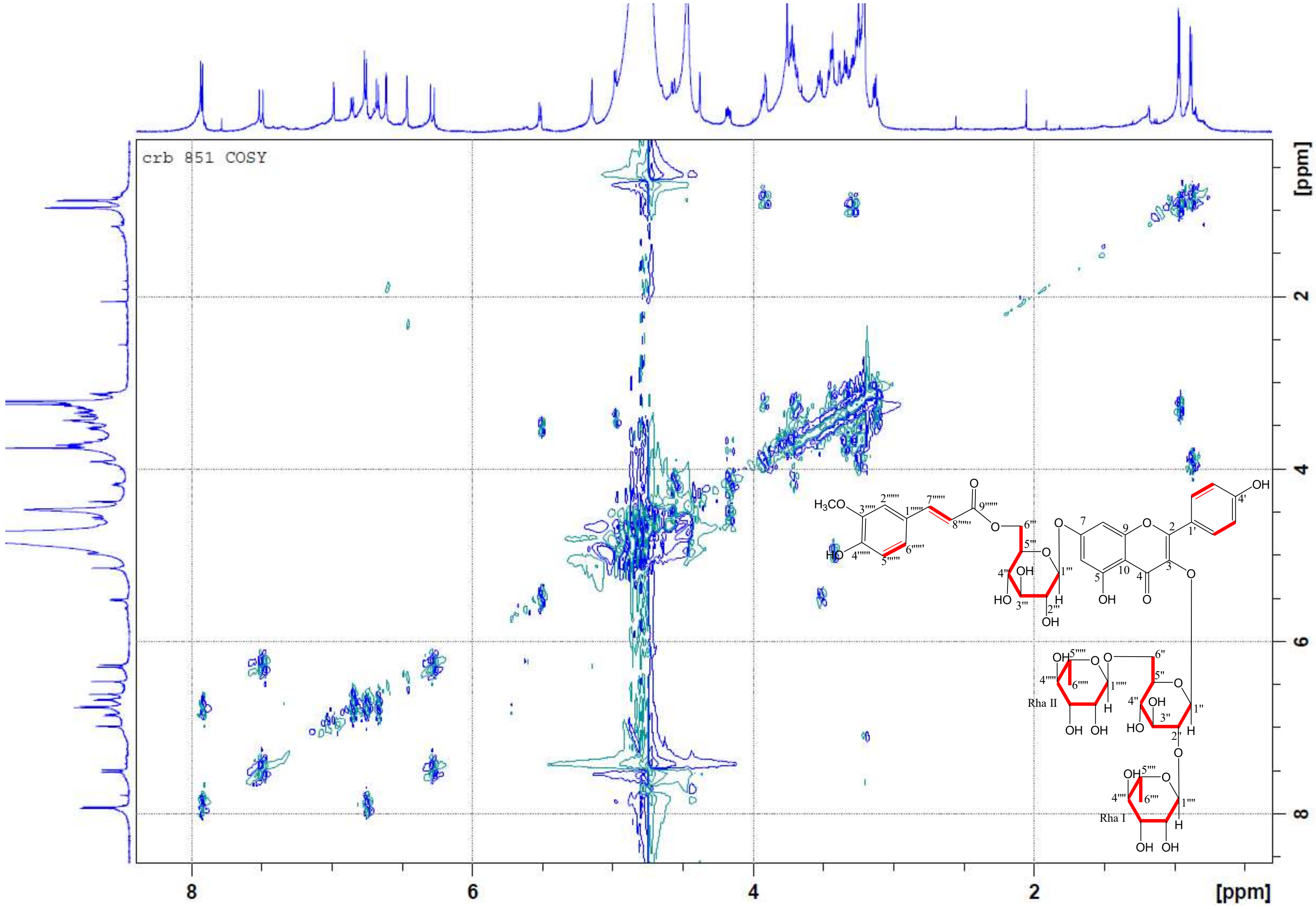

Figure S24. The HSQC spectrum of **3** in CD_3OD, 600 MHz.

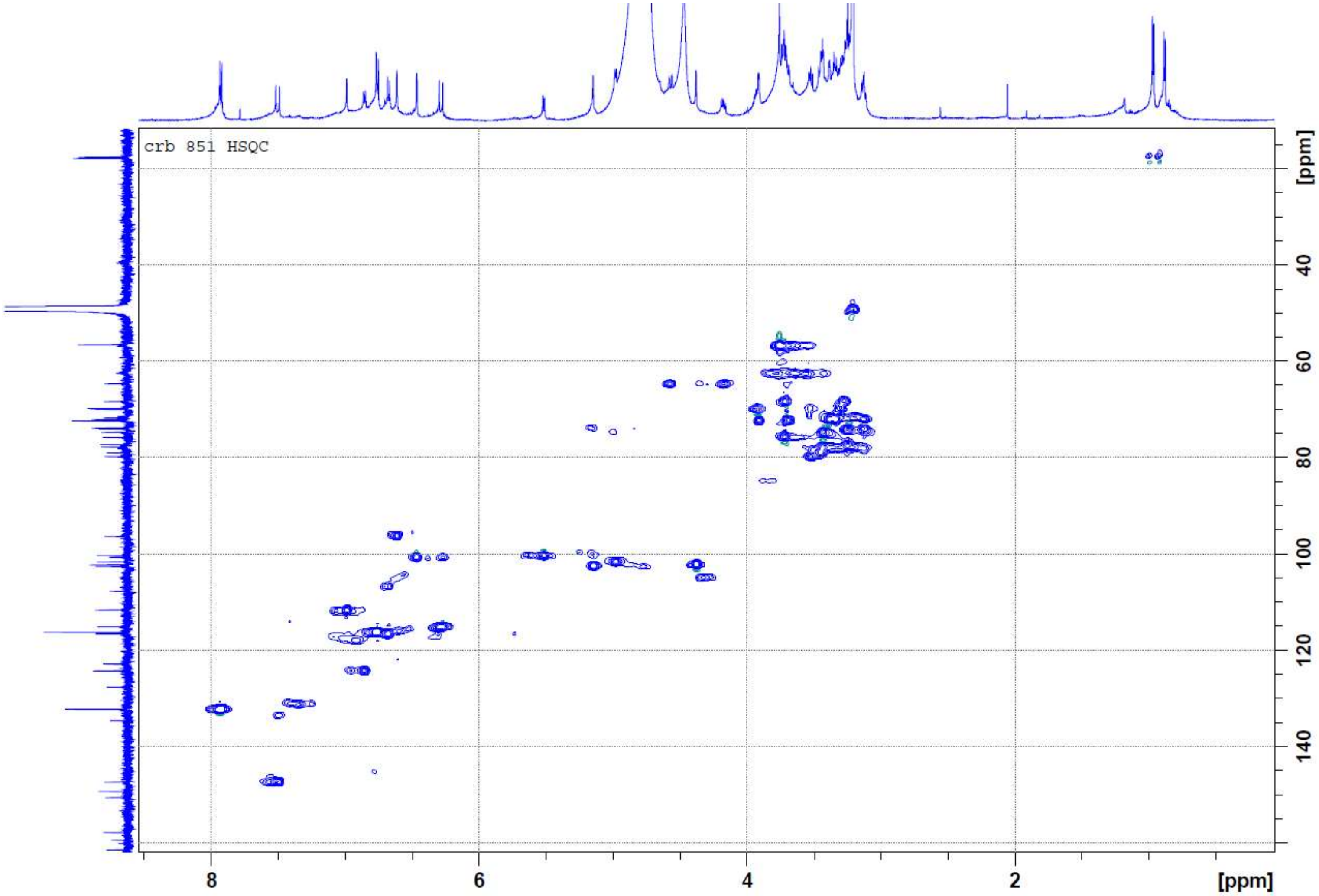

Figure S25. The HMBC spectrum of **3** in CD_3OD, 600 MHz.

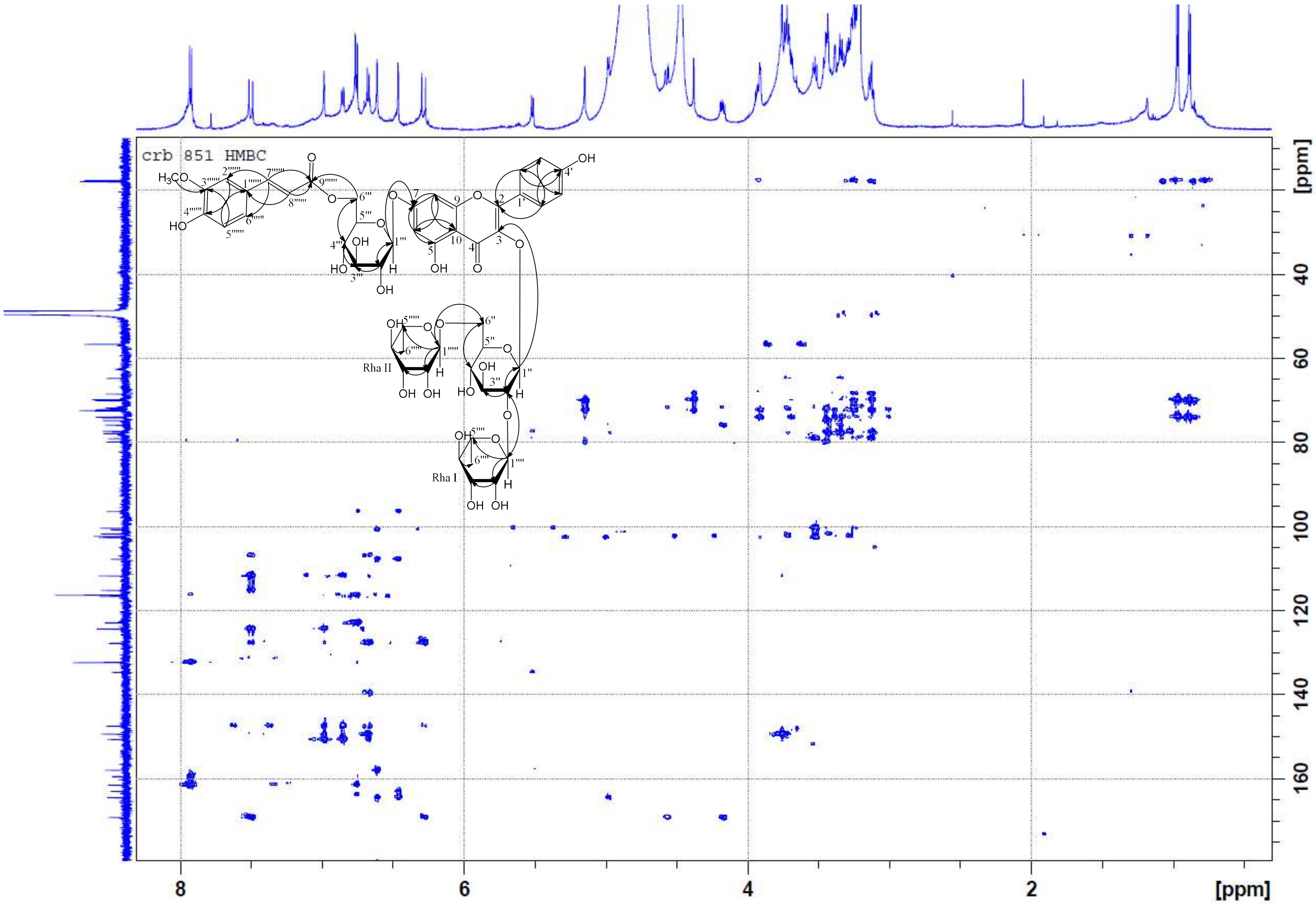

Figure S26. The UV spectrum of **3** in CH_3OH.

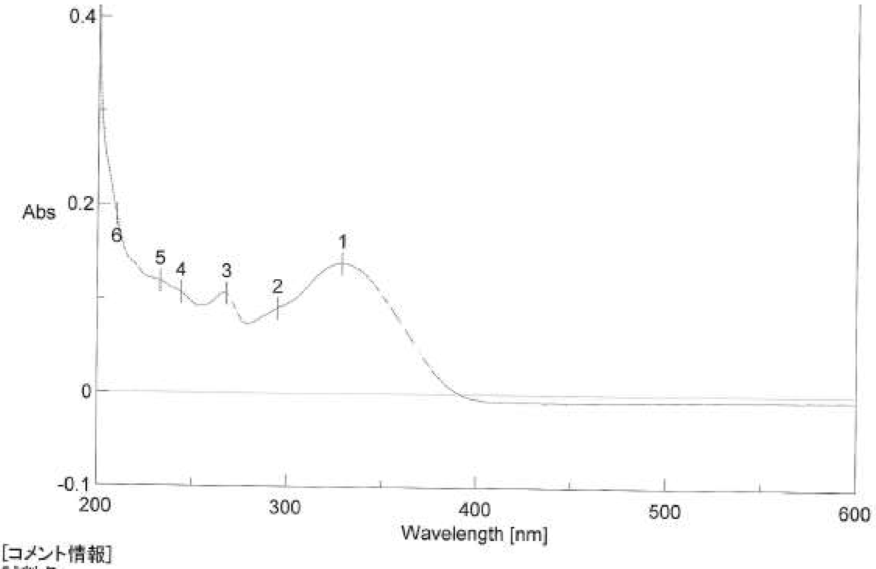

[コメント情報]
試料名
コメント
測定者
所属
会社　HU

[データ情報]

作成日時	2018/07/02 20:37
データタイプ	等間隔データ
横軸	Wavelength [nm]
縦軸	Abs
スタート	600 nm
エンド	200 nm
データ間隔	1 nm
データ数	401

[測定情報]

装置名	V-630
機種名	V-630
シリアル番号	A042161148
測定日時	2018/07/02 20:37
測光モード	Abs
測定範囲	600 - 200 nm
データ取込間隔	1 nm
UV/Vis バンド幅	1.5 nm
レスポンス	Fast
走査速度	400 nm/min
光源切換	340 nm
光源	D2/WI
フィルタ切換	ステップ
補正	ベースライン

[ピーク検出結果]

No.	位置	強度	No.	位置	強度
1	329	0.138572	2	295	0.0901297
3	268	0.105603	4	244	0.106448
5	233	0.118901	6	210	0.18853

Figure S27. The IR spectrum of **3**.

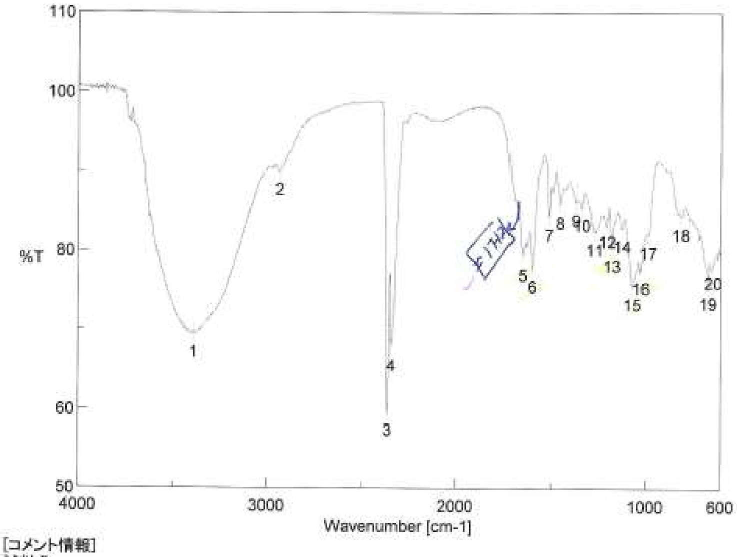

[コメント情報]
試料名
コメント
測定者
所属
会社 HU

[データ情報]		[測定情報]	
作成日時	2018/06/28 17:19	機種名	FT/IR-4600typeA
		シリアル番号	D015461786
データタイプ	等間隔データ		
横軸	Wavenumber [cm-1]	測定日時	2018/06/28 17:19
縦軸	%T		
スタート	599.753 cm-1	光源	標準光源
エンド	7800.65 cm-1	検出器	TGS
データ間隔	0.964233 cm-1	積算回数	10
データ数	7469	分解	4 cm-1
		ゼロフィリング	On
		アポダイゼーション	Cosine
		ゲイン	Auto (2)
		アパーチャー	Auto (7.1 mm)
		スキャンスピード	Auto (2 mm/sec)
		フィルタ	Auto (30000 Hz)

[ピーク検出結果]

No.	位置	強度	No.	位置	強度
1	3389.28	69.5281	2	2932.23	89.9835
3	2362.37	59.6281	4	2341.16	67.8766
5	1651.73	79.3603	6	1601.59	77.9251
7	1513.85	84.4268	8	1455.03	85.9357
9	1371.14	86.2565	10	1341.25	85.6483
11	1271.82	82.553	12	1205.29	83.5948
13	1180.22	80.5581	14	1126.22	83.012

[ピーク検出結果]

No.	位置	強度	No.	位置	強度
15	1071.26	75.6865	16	1024.98	77.7535
17	987.375	82.1855	18	814.777	84.4895
19	669.178	75.8501	20	641.215	78.4624

Figure S28. The HRESIMS spectrum of **4**.

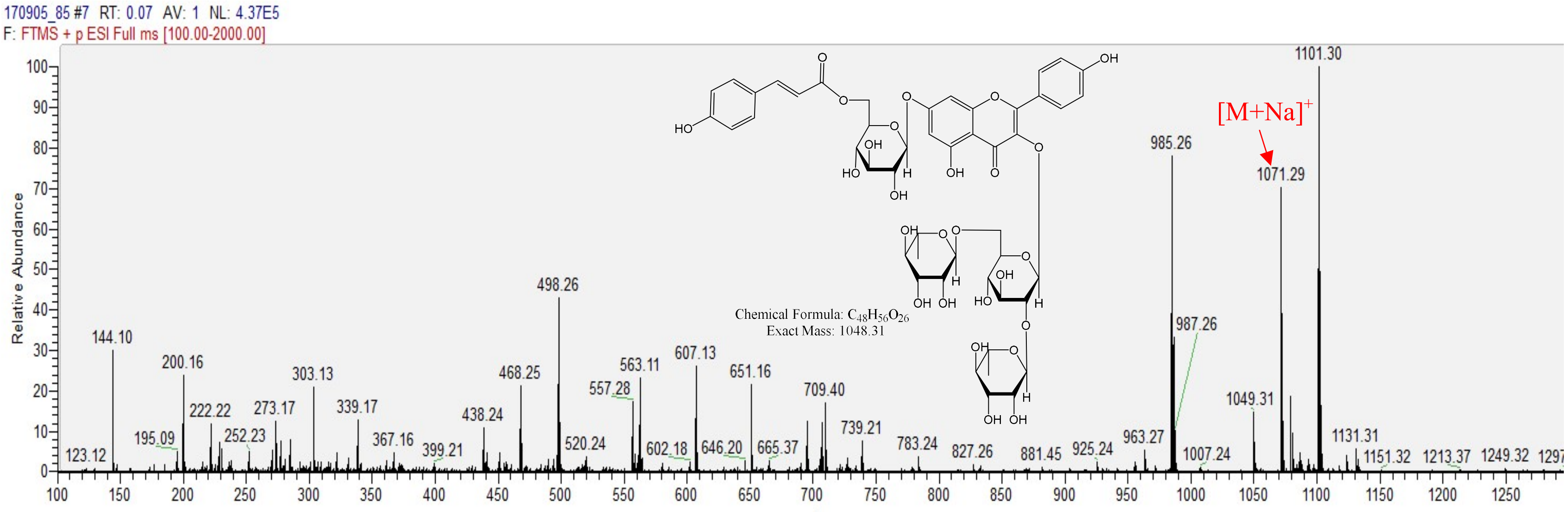

Elemental composition search on mass 1071.29

m/z= 1066.29-1076.29

m/z	Theo. Mass	Delta (mmu)	RDB equiv.	Composition
1071.2925	1071.2952	-2.72	20.5	$C_{48}H_{56}O_{26}Na$

Figure S29. The ^{1}H NMR spectrum of **4** in CD_3OD, 600 MHz.

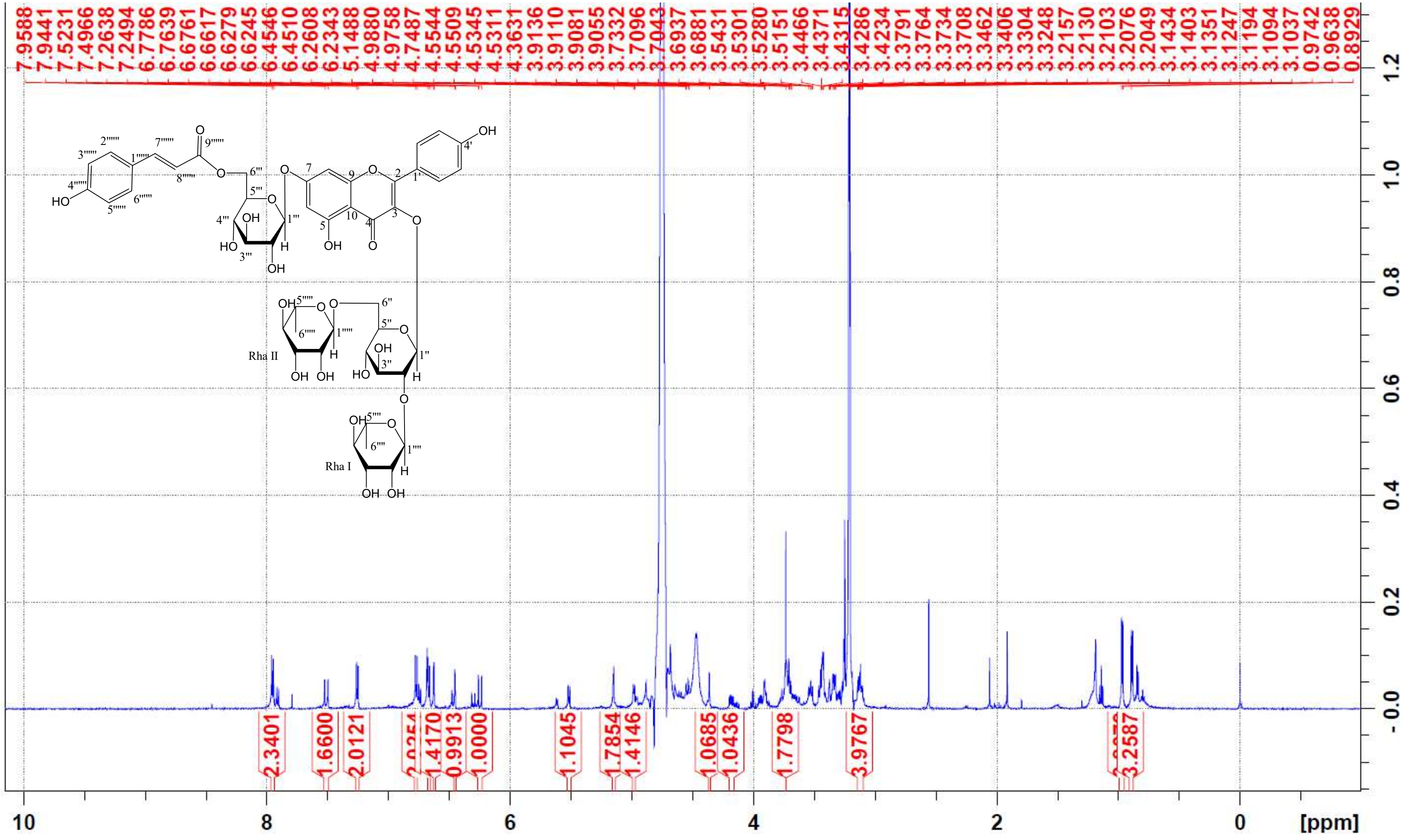

Figure S30. The ^{13}C NMR spectrum of **4** in CD_3OD, 150 MHz.

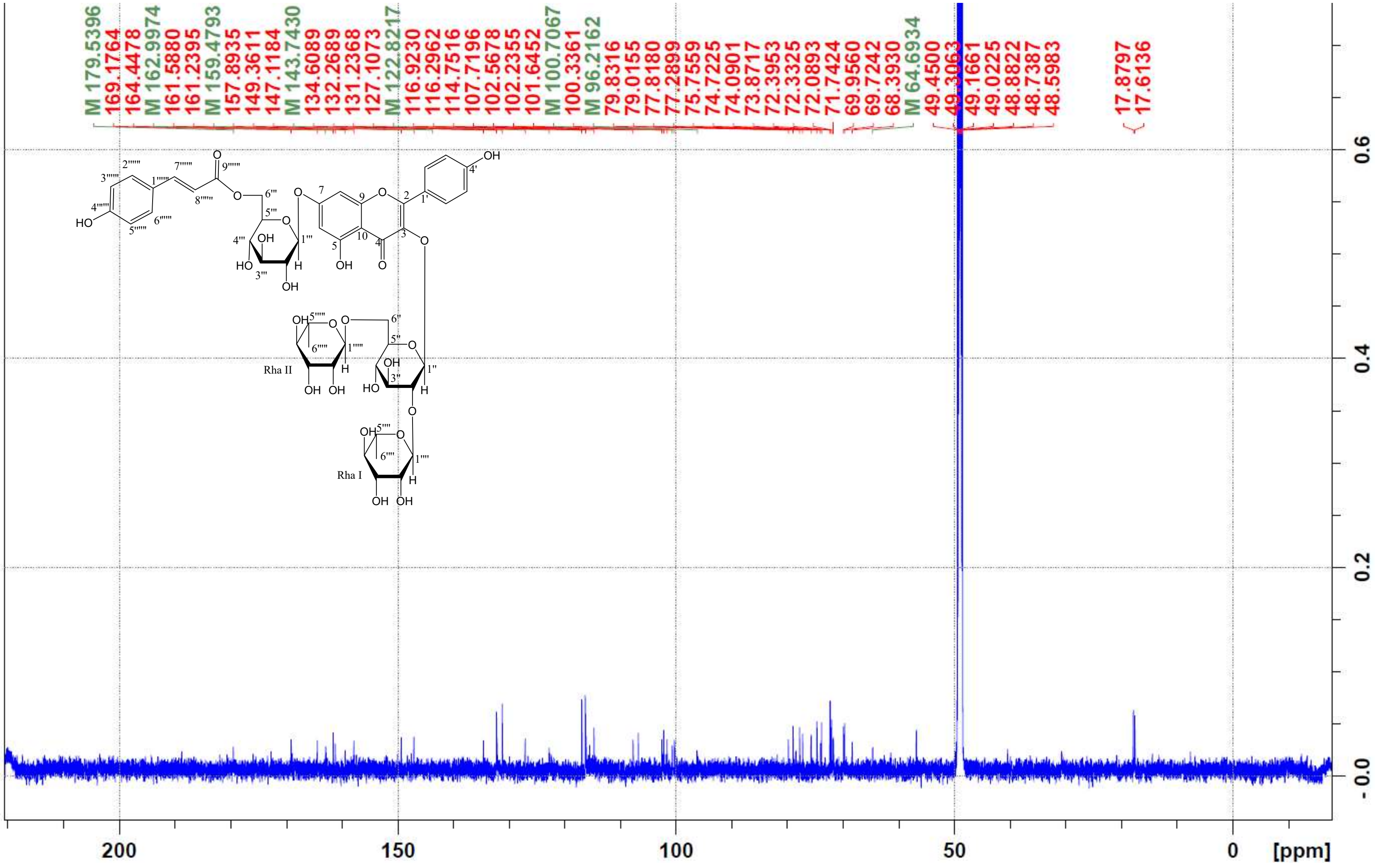

Figure S31. The COSY NMR spectrum of **4** in CD_3OD, 600 MHz.

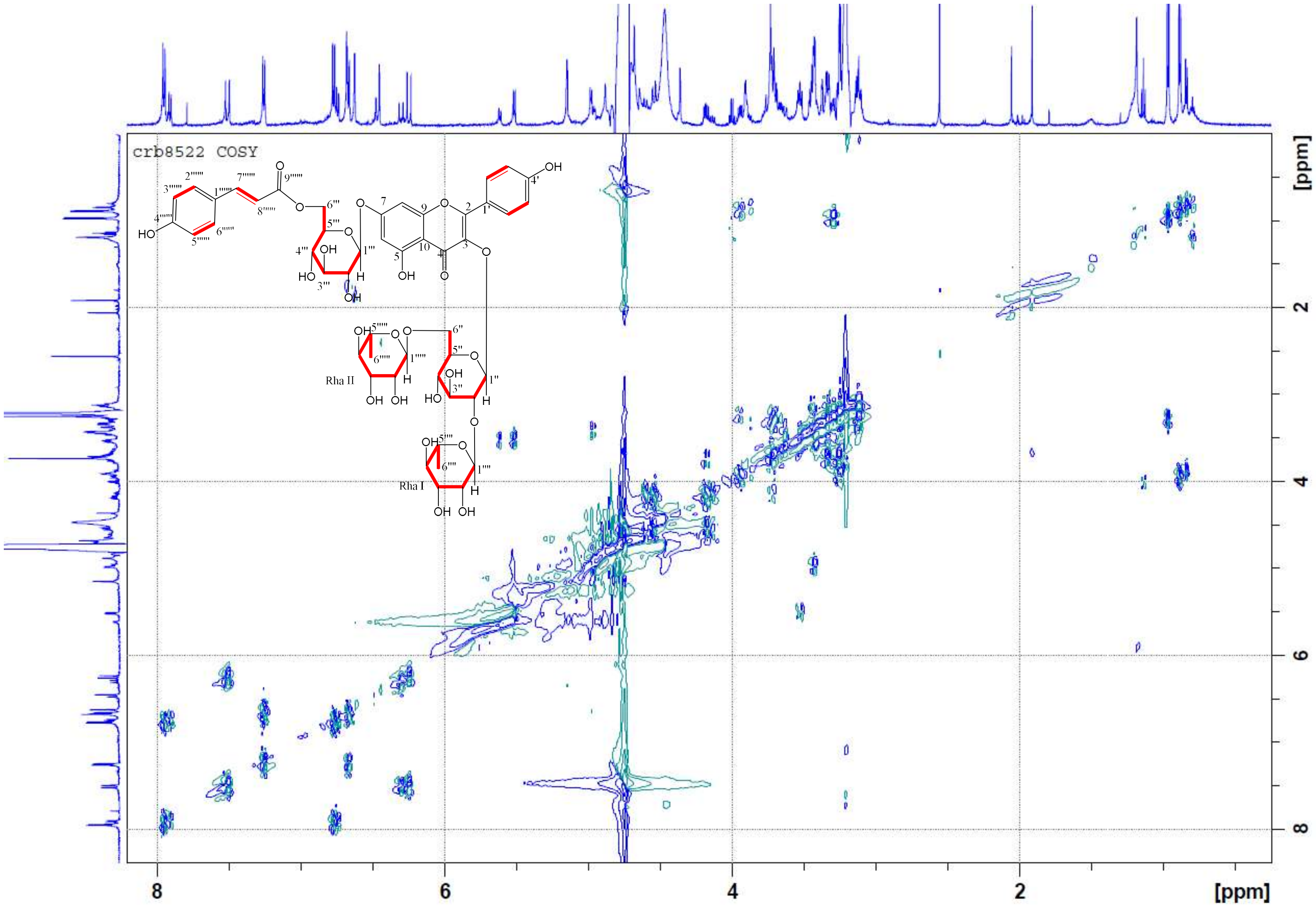

Figure S32. The HSQC NMR spectrum of **4** in CD_3OD, 600 MHz.

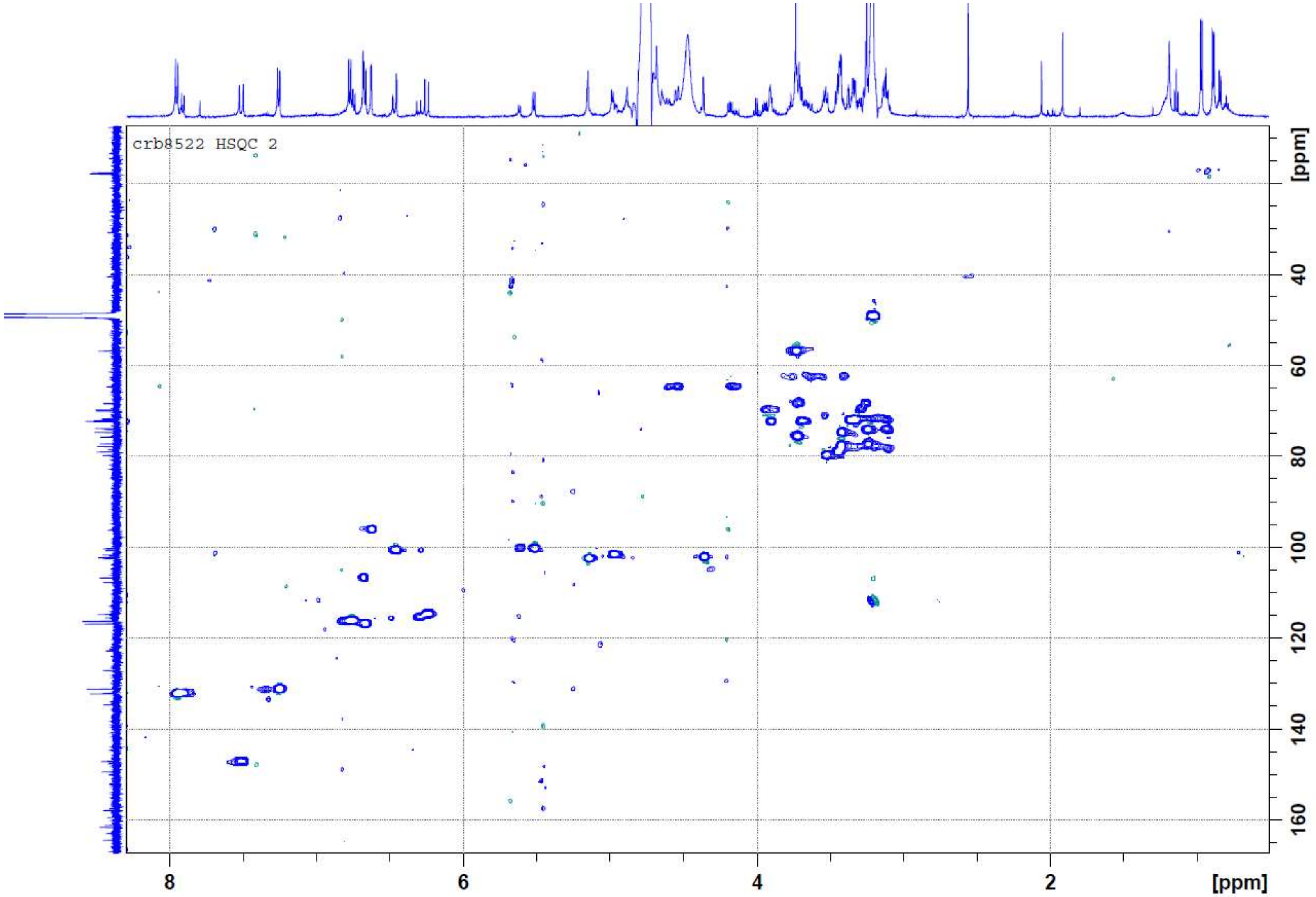

Figure S33. The HMBC NMR spectrum of **4** in CD_3OD, 600 MHz.

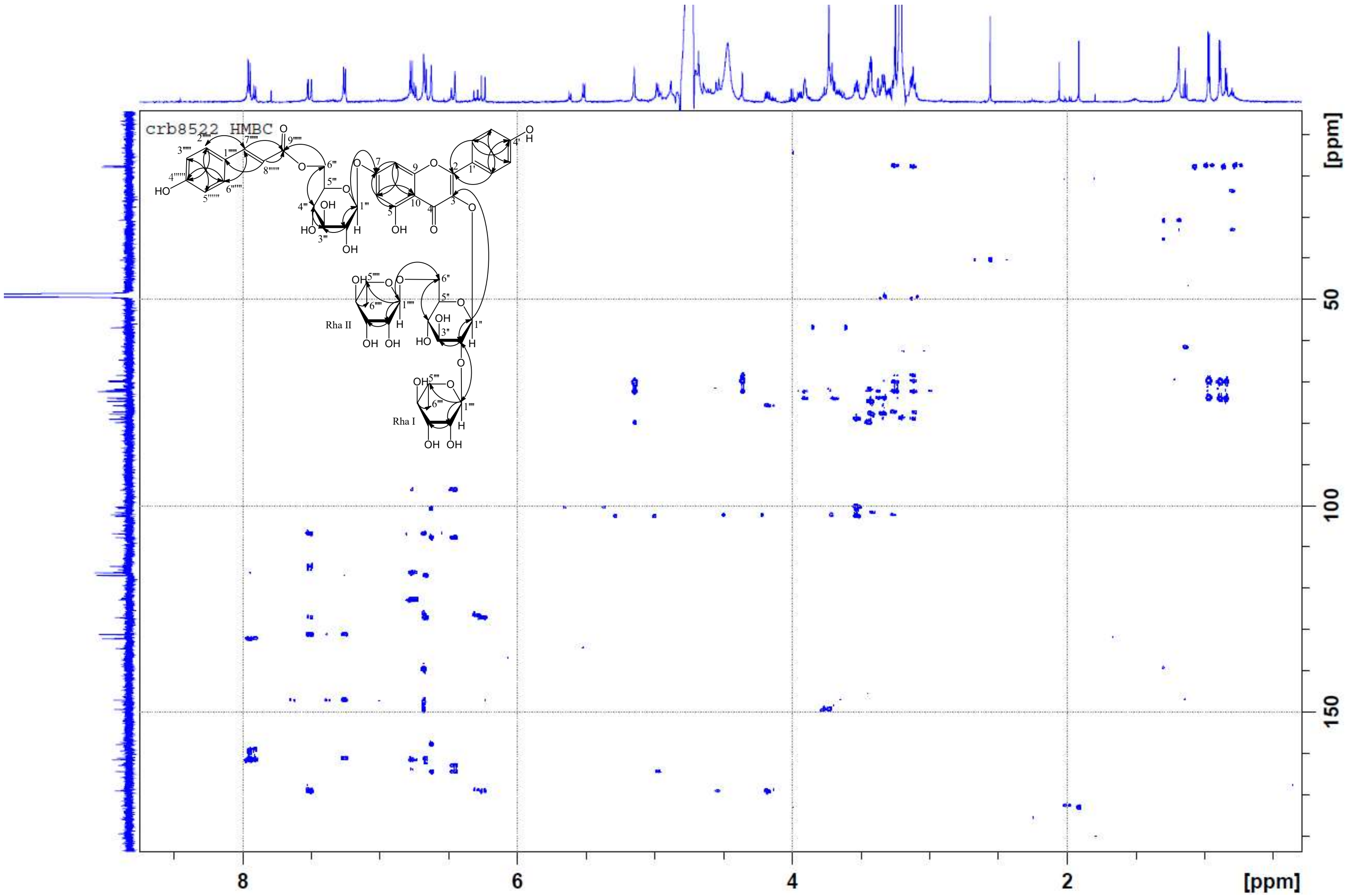

Figure S34. The UV spectrum of **4** in CH_3OH.

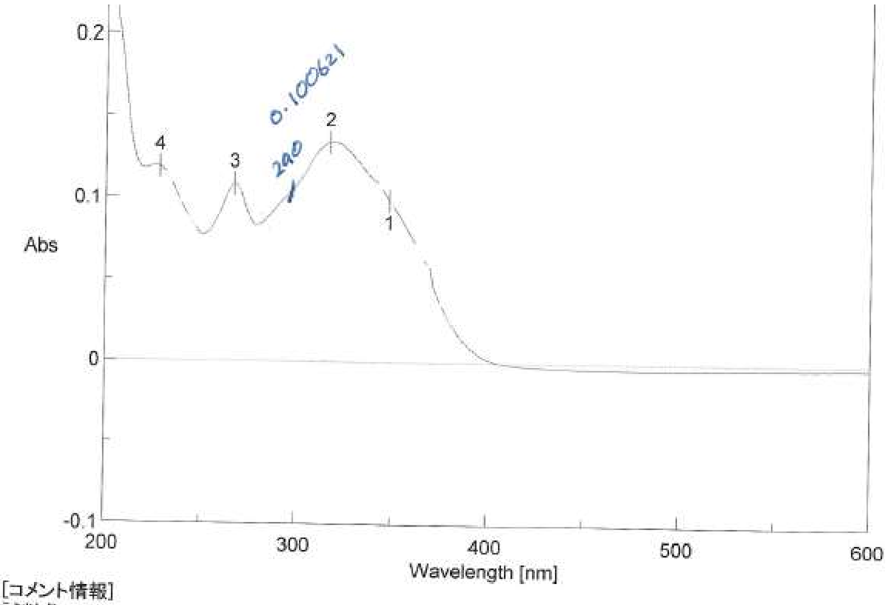

[コメント情報]

試料名	
コメント	
測定者	
所属	
会社	HU

[データ情報]

作成日時	2018/07/04 18:26
データタイプ	等間隔データ
横軸	Wavelength [nm]
縦軸	Abs
スタート	600 nm
エンド	200 nm
データ間隔	1 nm
データ数	401

[測定情報]

装置名	V-630
機種名	V-630
シリアル番号	A042161148
測定日時	2018/07/04 18:26
測光モード	Abs
測定範囲	600 - 200 nm
データ取込間隔	1 nm
UV/Vis バンド幅	1.5 nm
レスポンス	Fast
走査速度	400 nm/min
光源切換	340 nm
光源	D2/WI
フィルタ切換	ステップ
補正	ベースライン

[ピーク検出結果]

No.	位置	強度	No.	位置	強度
1	348	0.0992154	2	317	0.134589
3	267	0.108621	4	228	0.118853

Figure S35. The IR spectrum of **4**.

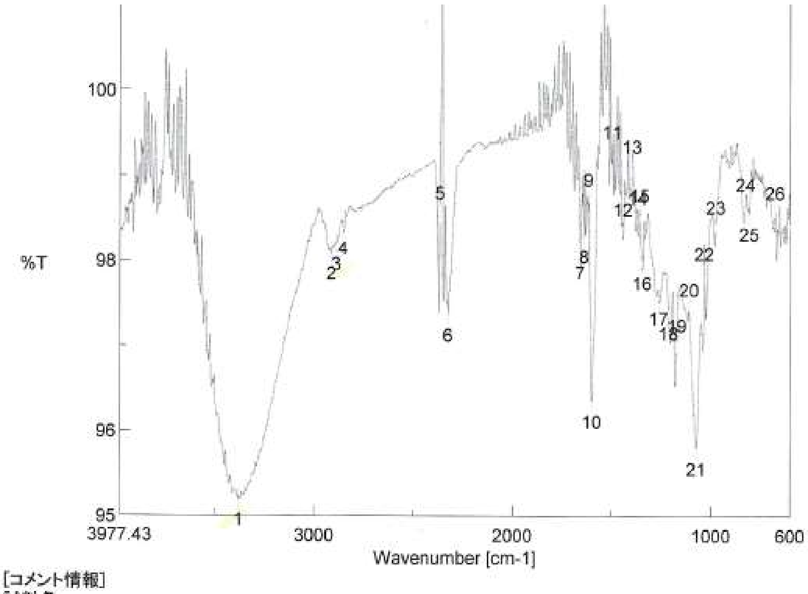

[コメント情報]

試料名	
コメント	
測定者	
所属	
会社	HU

[データ情報]

作成日時	2018/07/04 16:16
データタイプ	等間隔データ
横軸	Wavenumber [cm-1]
縦軸	%T
スタート	599.753 cm-1
エンド	7800.65 cm-1
データ間隔	0.964233 cm-1
データ数	7469

[測定情報]

機種名	FT/IR-4600typeA
シリアル番号	D015461786
測定日時	2018/07/04 16:16
光源	標準光源
検出器	TGS
積算回数	10
分解	4 cm-1
ゼロフィリング	On
アポダイゼーション	Cosine
ゲイン	Auto (2)
アパーチャー	Auto (7.1 mm)
スキャンスピード	Auto (2 mm/sec)
フィルタ	Auto (30000 Hz)

[ピーク検出結果]

No.	位置	強度	No.	位置	強度
1	3379.64	95.1948	2	2912.95	98.0898
3	2889.81	98.2016	4	2855.1	98.387
5	2367.19	98.5577	6	2325.73	97.3581
7	1660.41	98.1028	8	1641.13	98.2915
9	1617.98	98.7136	10	1601.59	96.3532
11	1499.38	99.2697	12	1445.39	98.3674
13	1399.1	99.1031	14	1372.1	98.5032

[ピーク検出結果]

No.	位置	強度	No.	位置	強度
15	1360.53	98.5325	16	1348.96	97.9792
17	1265.07	97.5596	18	1214.93	97.3881
19	1172.51	97.001	20	1110.8	97.4221
21	1074.16	95.7951	22	1034.62	97.8447
23	976.769	98.3952	24	826.348	98.6617
25	807.063	98.5589	26	676.892	98.5653
